# Supplementary material for: Prospective study of factors associated with asthma attack recurrence (ATTACK) in children from three Ecuadorian cities during COVID-19: a study protocol
Source: BMJ Open. 2022 Jun 16;12(6):e056295. doi: 10.1136/bmjopen-2021-056295 (PMC9207574; doi:10.1136/bmjopen-2021-056295)
Supplement: Author's manuscript [file bmjopen-2021-056295.draft_revisions.pdf]

BMJ Open is committed to open peer review. As part of this commitment we make the peer review history of every article we publish publicly available.

When an article is published we post the peer reviewers' comments and the authors' responses online. We also post the versions of the paper that were used during peer review. These are the versions that the peer review comments apply to.

The versions of the paper that follow are the versions that were submitted during the peer review process. They are not the versions of record or the final published versions. They should not be cited or distributed as the published version of this manuscript.

BMJ Open is an open access journal and the full, final, typeset and author-corrected version of record of the manuscript is available on our site with no access controls, subscription charges or pay-per-view fees (http://bmjopen.bmj.com).

If you have any questions on BMJ Open's open peer review process please email info.bmjopen@bmj.com

A prospective study of factors associated with asthma attack recurrence in children from three Ecuadorian cities during Covid-19: the Asthma Attacks study (ATTACK)

Journal:	*BMJ Open*
Manuscript ID	bmjopen-2021-056295
Article Type:	Protocol
Date Submitted by the Author:	11-Aug-2021

Complete List of Authors:	Morillo, Diana; Universidad Internacional del Ecuador<br>Mena-Bucheli, Santiago; Universidad Internacional del Ecuador<br>Ochoa, Angélica; Universidad de Cuenca<br>Chico, Martha E; Universidad Internacional del Ecuador<br>Rodas, Claudia; Universidad de Azuay<br>Maldonado, Augusto; Universidad San Francisco de Quito; Hospital General Docente Calderón<br>Arteaga, Karen; Hospital Verdi Cevallos Balda<br>Rodriguez, Alejandro<br>Figueiredo, Camila; Universidade Federal da Bahia<br>Ardura-Garcia, Cristina; University of Bern<br>Bachmann, Max; Univeristy of East Anglia, Norwich Medical School, University of East Anglia<br>Perkin, Michael; St George's, Population Health Research Institute<br>Chis Ster, Irina; St. George's University of London; St George's University of London<br>Cruz, Alvaro; Universidade Federal da Bahia<br>Romero, Natalia; Universidad Internacional del Ecuador, Quito, School of Medicine; Grups de Recerca d'America i Africa Llatines, GRAAL<br>Cooper, Philip; Universidad Internacional del Ecuador; St George's University of London

Keywords:	Asthma < THORACIC MEDICINE, COVID-19, Epidemiology < THORACIC MEDICINE

SCHOLARONE™
Manuscripts

Title: A prospective study of factors associated with asthma attack recurrence in children from three Ecuadorian cities during Covid-19: the Asthma Attacks study (ATTACK)

Authors

Diana Morillo[1], Santiago Mena-Bucheli[1], Angélica Ochoa[2], Martha E Chico[1], Claudia Rodas[3], Augusto Maldonado [4,5], Karen Arteaga[6], Alejandro Rodriguez[1], Camila Figueiredo[7], Cristina Ardura-Garcia[8], Max Bachmann[9], Michael Perkin[10], Irina Chis Ster[10], Alvaro A Cruz[7, 11], *Natalia Romero[1,12], *Philip J Cooper [1,10]

Affiliations

[1] Universidad Internacional del Ecuador, Quito, Ecuador.

[2] Universidad de Cuenca, Cuenca, Ecuador.

[3] Universidad de Azuay, Cuenca, Ecuador

[4] Universidad San Francisco de Quito, Quito, Ecuador

[5] Hospital General Docente Calderón, Quito, Ecuador

[6] Hospital Verdi Cevallos Balda, Portoviejo, Ecuador

[7] Universidade Federal da Bahia, Salvador, Brazil

[8]Institute of Social and Preventive Medicine, University of Bern, Bern, Switzerland.

[9] University of East Anglia, Norwich, UK.

[10] St George's University of London, London, UK.

[11]ProAR-Center of Excellence in Asthma, Salvador, Brazil

[12]Grups de Recerca d'Amèrica i Àfrica Llatines GRAAL, Barcelona, Spain

*Contributed equally

Correspondence

Philip J. Cooper, Escuela de Medicina, Universidad Internacional del Ecuador, Av. Jorge Fernández y Av. Simon Bolivar, Quito, Ecuador.

Email: pcooper@sgul.ac.uk

Word count

Abstract: 282
Text: 3,782

Strengths and limitations of this study

- Asthma attacks are a growing cause of morbidity in children and burden on health systems in low and middle-income countries, and there is limited information on factors associated with asthma attacks in poor urban settings in Latin America.
- This protocol describes a study designed to investigate risk factors associated with recurrence of asthma attacks in children and adolescents living in marginalized settings in three Ecuadorian cities.
- This study is recruiting children and adolescents with uncontrolled asthma symptoms and asthma attacks and following them prospectively for 6 to 12 months to identify factors associated with recurrence of attacks in the context of the Covid-19 pandemic.
- The Covid-19 pandemic has had a major impact on health seeking behaviours while mitigation strategies to control transmission of the novel coronavirus, SARS-CoV-2, have resulted in marked declines in the circulation of respiratory viruses that are considered to underlie a high proportion of asthma attacks. Such factors may interfere with recruitment and follow-up of study participants.

For peer review only - http://bmjopen.bmj.com/site/about/guidelines.xhtml

Abstract

Introduction: Asthma is a growing health problem in children living in marginalized urban settings in low and middle-income countries. Asthma attacks are an important cause of emergency care attendance and long-term morbidity. We designed a prospective study, the Asthma Attacks study (ATTACK), to identify factors associated with recurrence of asthma attacks among children and adolescents attending emergency care in three Ecuadorian cities.

Methods and analysis: Prospective clinical cohort study designed to identify risk factors associated with recurrence of asthma attacks (or exacerbations) in 450 children and adolescents aged 5 to 17 years attending emergency care in public hospitals in three Ecuadorian cities (Quito, Cuenca, and Portoviejo). The primary outcome will be rate of asthma attack recurrence during 6 to 12 months of follow-up. Data are being collected at baseline and during follow-up by questionnaire: sociodemographic data, asthma history and management (baseline only); recurrence of asthma symptoms and attacks (monthly); economic costs of asthma to family; Asthma Control Test; Pediatric Asthma Quality of life Questionnaire; and Newcastle Asthma Knowledge Questionnaire (baseline only). In addition, the following are being measured at baseline and during follow-up: lung function and reversibility by spirometry before and after salbutamol; fractional exhaled nitric oxide (FeNO); and presence of IgG antibodies to SARS-CoV-2 in blood. Recruitment started in 2019 but because of severe disruption to emergency health services caused by the Covid-19 pandemic, eligibility criteria were modified to include asthmatic children with uncontrolled symptoms and registered with collaborating hospitals. Data will be analysed using logistic regression and survival analyses.

Ethics and dissemination: The study results will be disseminated through presentations at conferences and to key stakeholder groups including policy makers, postgraduate student theses, peer-review publications, and on a study website.

Introduction

Asthma is the most common chronic disease of childhood and is estimated to affect more than 350 million people worldwide [1]. Although initially described as a disease of high-income countries (HICs) such as the United Kingdom, international comparisons using standardized methodologies have shown a similar or greater prevalence among children in urban centres in Latin America as observed in HICs [2]. There is evidence that the prevalence of asthma has increased in low- and middle-income countries (LMICs) over recent decades [2].

Asthma attacks or exacerbations are frequent in children and are a common cause of hospitalization [3], most frequently associated with respiratory viral infections [4], but also with exposures to allergens [5] and air pollution [6] among individuals with an underlying genetic susceptibility [7]. Asthma is an underdiagnosed disease because of lack of specialized personnel and resources to evaluate lung function. Further, limited access to specialized care and treatment [8], accompanied by poor treatment adherence [9,10] are important contributing factors to inadequate disease control and attacks, especially in underprivileged populations. In such settings, asthma patients with poor control of their daily symptoms often rely on emergency room care for management of their symptoms [11], resulting in high economic costs to health systems and patients' families [8,12].

To understand better factors contributing to the risk of asthma attacks, it is important identify those factors contributing to suboptimal management which can be improved through better health care [13]. Several studies have investigated potential predictors of recurrence of asthma attacks among children attending emergency services. Factors identified as important included a history of previous emergency attendances for acute asthma attacks, younger age [13–15], ethnicity of African descent [16,17], and low socioeconomic status [13,18]. These studies were conducted almost exclusively in North America and frequently included adult Hispanic populations living there. There are relatively few such studies done in LMICs [13,16,18].

For peer review only - http://bmjopen.bmj.com/site/about/guidelines.xhtml

The Asthma Attacks Study (ATTACK) aims to identify factors related to recurrence of asthma attacks in Ecuadorian children attending emergency care in three Ecuadorian cities. Recruitment into the study started in March 2019 but was interrupted by the Covid-19 pandemic that had a major impact on use of and access to emergency care [19]. In consequence, the original protocol was modified to allow continued recruitment and measure the impact of the Covid-19 pandemic and exposures to SARS-CoV-2 on asthma attacks in children and adolescents.

What is known about factors related to asthma attacks and impact of COVID-19 pandemic?

Severe asthma in children is associated with loss of lung function [20], as well as with high costs of medical and family care [2]. In Latin America, asthma has been described as a major public health problem because of a high prevalence and significant associated morbidity and mortality [21]. Asthma control among children in Latin America has been reported to be among the worse worldwide [22–25]. The Latin America Asthma Insights and Management study reported adequate asthma control in less than 20% of asthmatic children in Argentina, Brazil, Mexico, Venezuela, and Puerto Rico [22]. A case-control study of asthmatic children with an acute attack attending an Emergency Room (ER) in coastal Ecuador showed that, over the previous 12 months, only 20% of children had visited a doctor in the past year, 86% had attended an emergency room at least once prior, and none were receiving inhaled corticosteroids despite over half having suffered four or more acute exacerbations [26]. A previous prospective study of children with asthma attacks in coastal Ecuador showed a recurrence rate of 46% within 6 months [13].

Asthma attacks represent an acute or subacute increase in respiratory symptoms [25] and severe attacks - defined as requiring ER or hospital care and treatment with systemic corticosteroids for at least 3 days - are associated with loss of lung function [20] and high economic costs to the patient’s family and health care system [7], as well as absenteeism in work and school.

Asthma attacks may be prevented either by avoiding previously identified triggers or by appropriate preventive treatment. Patients using inhaled corticosteroids (ICS) were 3 times more likely to have well-controlled asthma [22], a reduction in symptoms independent of frequency, and better lung function [1]. In Brazil, preventive strategies have been successfully implemented through asthma control programs such as Pro-AR in Salvador de Bahía to reduce future risk in asthmatic patients from less privileged backgrounds, through the provision of ICS [27]. There has been a shift to using ICS more widely for control of asthma, for example, through the provision of combination inhalers including both ICS and a beta$_2$ agonist for moderate to severe asthma [28]. More recent evidence indicate that the use of ICS may be beneficial even for asthmatic children with mild disease that accounts for 30-40% of severe attacks [29]. Treatment recommendations for children and adolescents with mild disease are now shifting to use of ICS (plus long-acting beta-agonists in combination inhalers) as required [30,31]. Despite such initiatives, access to ICS in many LMIC settings is limited within health care systems because of cost and a failure to recognize the public health impact of childhood asthma. In such settings, children with asthma symptoms frequently over-rely on ER care for the acute control of their symptoms in the absence of adequate provision of specialized care for their long-term management [26,32].

Soon after the emergence of the novel coronavirus, SARS-CoV-2, in humans and its spread into a global pandemic, the question was raised as to whether this novel viral infection might alter the risk of asthma symptoms because of deficient antiviral immune responses and the tendency to increase exacerbations during infections with other respiratory viruses [33]. To date, evidence from studies of asthmatic children and adults do not indicate an increased risk [34,35]. Characteristics of asthmatic airways among those with T2-linked phenotypes may reduce the expression of the cellular receptor for SARS-CoV-2, angiotensin-converting enzyme 2 (ACE2), in the airways [36]. It has been suggested that the use of ICS among asthmatics may reduce risk of severe Covid-19 by suppressing inflammation and enhancing antiviral defenses [37], although the evidence remains unclear [38] and there are very limited data from pediatric populations [38]. The Covid-19 pandemic has resulted in marked reductions in hospital

For peer review only - http://bmjopen.bmj.com/site/about/guidelines.xhtml

visits including to emergency rooms (Figure 1), reduced attack rates, and better lung function among children with asthma [19,39], which are likely consequences of mitigation measures to reduce transmission of SARS-CoV-2 (such as lockdowns, face masks, and social distancing) that have resulted also in the reduced transmission of other respiratory viruses [4,6,10,39]. Treatment adherence may have improved also because of better parental supervision of asthma medications [40]. There are limited data on the impact of exposure to SARS-CoV-2 on asthma attacks and uncontrolled symptoms and the impact of the collateral effects of the Covid-19 pandemic including reduced access to health services and medications and effects of Covid-19 mitigation efforts in resource-poor LMIC settings.

Figure 1: Impact of the COVID-19 pandemic in the 3 study centres in Quito, Portoviejo and Cuenca, Ecuador, on recruitment rates into the study and number of potentially eligible subjects identified in collaborating emergency rooms. Red bars show numbers recruited as a proportion (%) of those eligible (blue bars) prior to a legally enforced national lockdown on 16th March 2020.

Methods and analysis

The Asthma Attack study

A prospective multi-center study is being done in children aged 5 to 17 years in low-income settings in three Ecuadorian cities (Quito, Cuenca, and Portoviejo) to study factors associated with acute asthma attacks. Because of the impact of the Covid-19 pandemic on risk of asthma attacks and health care access, the study was divided into 2 phases of recruitment and follow-up: 1) Phase I (or pre-Covid-19 from May 2019 to March 2020) evaluated risk factors associated with recurrence of acute asthma attacks among children and adolescents attending emergency rooms of public hospitals in low-income settings in the three cities; and 2) Phase II (or peri-COVID-19 from March 2021 to March 2022) is evaluating risk factors associated with attacks among children and adolescents with asthma and current symptoms who have previously attended specialist care at public hospitals in Cuenca and Portoviejo. Phase II is also evaluating the effects of exposures to SARS-CoV-2 on asthma attacks and symptoms. In addition,

both study phases are evaluating the impact of asthma, and adequate management and control of asthma, on the quality of life and economic costs for the family of children and adolescents with asthma. Qualitative studies of health worker's perspectives on asthma care coordination between primary and specialized health care are being done also as described elsewhere.

Study design and setting

This is a multi-center prospective study conducted in three Ecuadorian cities (Quito, Cuenca, and Portoviejo). Quito, the capital, and Cuenca, the third largest city, are located in the Andean highlands (altitude >2,500m) where the annual average temperature is ~16°C, while Portoviejo is in the coastal region (at sea level) with an annual average temperature of ~25 °C [41]. According to the 2010 census, the populations of the three cities are: Quito >2 million, Cuenca 0.5 million, and Portoviejo 0.28 million inhabitants. Ecuador is an upper-middle-income-country with a per capita income of $6,080 in 2019. The health system in Ecuador includes public and private provision. Public institutions offer universal health coverage that is stratified into different levels of care from primary to tertiary services [42]. Social security institutions offer health services to affiliated salaried workers and their families. The private sector includes for-profit entities (hospitals, clinics, dispensaries, doctor's offices, pharmacies, and prepaid health insurance companies) which are generally located in the main cities [43].

Study centers

The study was initially based in the tertiary care hospitals in each of the three cities (Hospital Docente General de Calderon, Quito; Hospital Vicente Corral Moscoso, Cuenca; and Hospital Verdi Cevallos Balda, Portoviejo).

Recruitment - Phase I (pre-COVID-19; May 2019 to March 2020)

Children and adolescents aged 5–17 years were either recruited, either while attending emergency rooms (ERs) with an asthma attack or through daily registries and contact telephone numbers of patients attending the ERs with an asthma attack. From May

For peer review only - http://bmjopen.bmj.com/site/about/guidelines.xhtml

2019, eligible patients (Table 1) were provided with a study information sheet on discharge and their parents were contacted to arrange an interview at a hospital consulting room 10-14 days later when they were recruited into the study after providing informed written parental consent and minor assent (8 years and older).

Recruitment – Phase II (peri-COVID-19; March 2021 to March 2022)

The Covid-19 pandemic had a dramatic impact on our ability to recruit children with asthma attacks in the three study hospitals that resulted in the suspension of recruitment in March 2020. This was owing to the reassignment of all 3 hospitals to Covid-19-only activities for prolonged periods and the imposition of a legally enforced lockdown nationally. Figure 2 shows the number of potentially eligible children identified in the 3 study hospitals by month in the period prior to Covid-19 and during the pandemic. There was as a marked reduction in eligible children attending the ERs immediately following the imposition of lockdown on 16th March 2020 and in the following 8 months. In the period following the initial lockdown and other mitigation strategies to reduce transmission of SARS-CoV-2, within the sample of asthmatic children recruited into Phase I of the study, there was evidence of dramatic reduction in health service attendance rather than changes in the incidence of attacks [19]. To allow continued recruitment, a second phase (Phase II) of the study was started with eligibility criteria modified to include children and adolescents aged 5-17 years registered as having asthma in patient registries at the study hospitals in Cuenca and Portoviejo (Quito was not included in Phase II because of logistic issues in that specific setting). The child's parent was contacted by telephone and asked about the child's current asthma symptoms. Children with current symptoms were considered eligible. Eligibility criteria for Phase I and II are shown in Table 1.

Table 1 Eligibility criteria for entry into Phase I and Phase II of study

Characteristic	Phase I	Phase II
Setting/study population	Public hospital ERs in Quito, Cuenca, and Portoviejo	Public hospital registry of patients with asthma in Cuenca and Portoviejo

For peer review only - http://bmjopen.bmj.com/site/about/guidelines.xhtml

Inclusion criteria	1. Aged 5-17 years<br>2. **Acute asthma attack attending ERs at public hospitals**<br>3. Living within 12 km of public hospital<br>4. Informed written consent from parents<br>5. Minor assent from children >=8 years	1. Aged 5-17 years<br>2. **Wheeze within the last 6 months**<br>3. Living within 12 km of public hospital<br>4. Informed written consent from parents<br>5. Minor assent from children >=8 years
Exclusion criteria	1. Other chronic disease<br>2. Living >12 km from public hospital	1. Other chronic disease<br>2. Living >12 km from public hospital

Data collection

A study manager supervised day-to-day activities of a dedicated teams (physician and nurse) of trained study personnel based in each of the public hospitals. Each participant was followed up for 12 months in Phase I and at least 6 months in Phase II. Standardized procedures were used throughout (Figure 2). Subjects were evaluated at monthly intervals using face-to-face and telemonitoring for follow-up. Both phases include face-to-face evaluations (evaluations at baseline, 2, 4, 6, 9, and 12 months in Phase I; baseline, 6, and 12 months in Phase II). Between March 2020 and March 2021, all face-to-face evaluations in Phase I were changed to telemonitoring apart from the final evaluation at 12 months which remained face-to-face, where possible. Final face-to-face evaluations were done in March-April 2021 on participants who had completed 12 months of follow-up between June 2019 and February 2020. The site of face-to-face evaluations was changed to either a clinic or home visit depending on the preference of the child's parents. Study procedures were the same in both phases of the study (Figure 2) with the following exceptions: 1) procedures requiring face-to-face evaluations at 6 and 12 months in Phase I (C-ACT, and PAQLQ) were dropped in Phase II and the remaining instruments were administered by telemonitoring; 2) blood samples (baseline, 6, and 12 months) are being collected from those recruited into Phase II of the study for measurement of anti-SARS-CoV-2 IgG antibodies; and 3)

For peer review only - http://bmjopen.bmj.com/site/about/guidelines.xhtml

measurement of lung function and fractional exhaled nitric oxide was suspended from March 2020 because of risks from potentially hazardous infectious aerosols until a time when this procedure can be done safely. Medications (inhaled salbutamol and fluticasone) were provided free of charge to participants when prescribed by a hospital physician and there was no medication in the hospital pharmacy. The study team ensured adequate inhaler technique during face-to-face clinic or home visits. Study personnel did not interfere with treatment indications of hospital staff.

Figure 2. Study procedures during baseline evaluation and follow-up in Phases I (A) and II (B) of study.

BaselineQ – General questionnaire based on Phase II of the International Study of Asthma and Allergies in Childhood; AFCQ- Asthma Family Costs Questionnaire; NAKQ – Newcastle Asthma Knowledge Questionnaire; ACT – Asthma Control Questionnaire (>11 years); C-ACT – Child Asthma Control Questionnaire (>=12 years); ARQ – Asthma Recurrence Questionnaire; FeNO – fractional exhaled nitric oxide. Follow-up in Phase I was for 12 months and in Phase II for a minimum of 6 months.

Questionnaires at baseline and follow-up

The following questionnaires were used as indicated in Figure 2; i) asthma questionnaire to collect information on history of asthma symptoms, treatment and management, and potential risk factors for asthma attacks. This questionnaire has been previously adapted from ISAAC Phase II and extensively field-tested for children and adolescents with asthma [13,26]; ii) Asthma Recurrence Questionnaire (ARQ) - questionnaire to monitor recurrence of asthma symptoms and monitor treatment during follow-up [13], and including symptoms and diagnosis of Covid-19 in Phase II; iii) Childhood Asthma Control Test (C-ACT) [44], completed by the child and parent for children aged <12 years or the ACT [45] for those aged ⩾12 years; iv) Paediatric Asthma Quality of Life Questionnaire (PAQLQ) [46]; v) Newcastle Asthma Knowledge Questionnaire (NAKQ) [47,48] validated in a Spanish-speaking populations; and vi)

Asthma Family Cost Questionnaire (AFCQ) [49]. Data collection is summarized in Table 2.

Table 2 Data collection at baseline and during follow-up in Phases I and II of study

Data collected	Baseline	Month 6	Month 12	Monthly
Asthma diagnosis	I/II			
History of asthma symptoms	I/II			
Current asthma symptoms	I/II	I/II	I/II	I/II
ER visits/ hospitalizations	I/II	I/II	I/II	I/II
Asthma medications	I/II	I/II	I/II	I/II
Asthma control (C-ACT/ACT)	I/II	(I)/II	(I)/II	
Asthma quality of life (PAQLQ)	I/II	(I)/II	(I)/II	
Asthma knowledge (NAKQ)	I			
Lung function<br>Reversibility<br>FeNO<br>Anti-SARS-CoV-2 IgG antibodies	I/(II)<br>I/(II)<br>I/II<br>II	(I/II)<br>(I/II)<br>(I)/II<br>II	(I)<br>(I)<br>(I)/II<br>II	
Covid-19 symptoms or diagnosis	II	II	II	II

FeNO – fractional exhaled nitric oxide. () – data collection where possible

Lung function test and Fractional Exhaled Nitric Oxide (FeNO)

Lung function testing is being done according to American Thoracic Society (ATS) criteria using a portable ultrasonic spirometer (EasyOne® device, ndd Medical Technologies). Global Lung Function Initiative (GLI-2012) reference values will be used

to calculate z-scores depending on age, sex, height, and ethnicity [50]. Lung function is being measured before and before and after 200 ug salbutamol administered through a spacer with a positive test for airways reactivity defined as an increase in FEV_1 of ≥12%. Fractional exhaled nitric oxide (FeNO) is being measured in parts per billion using NObreath (Bedfont Scientific, UK). As a consequence of lockdown and mitigation strategies to prevent transmission of SARS-CoV-2, measurement of lung function and FeNO was suspended in March 2020. FeNO measurement, a low effort procedure that carries a minimal risk of generating aerosols [51,52], was resumed in March 2021 for 12-month evaluations in Phase I and baseline evaluations in Phase II. FeNO is being done following the recommendations of the manufacturer to minimize potential risks [53]. Spirometry, which often induces cough, will be resumed, only if the procedure can be done without risk to staff, patients, and their families.

Blood samples

Blood sample are being taken at baseline, 6 and 12 months in Phase II. Blood is drawn from an antecubital vein into a 4 mL plastic tube containing EDTA as anticoagulant (Vacutainer, BD). After centrifugation, plasma is stored at -20C until analysis. Levels of IgG specific for SARS-CoV-2 are being measured using a validated enzyme linked immunosorbent assay [54].

Biosafety precautions relating to the COVID-19 pandemic

Personnel gathering data during face-to-face visits have been trained to use appropriate personal protective equipment according to international guidelines [55–57]. Immediately prior to a home or clinic visit, the child's carer is telephoned about potential symptoms or a COVID-19 diagnosis among family members or close contacts – if any symptoms are reported the visit is rescheduled after 2 weeks.

Sample size considerations

A sample size of 450 patients was proposed for Phase I, based on a binary outcome variable for recurrence of an asthma attack within 12 months, or not, with an estimated recurrence rate of about 50% during 6 months follow-up in a similar

population in Ecuador [13]. This sample size gives a power of 80% or more, based on having a minimum of 18 outcome events per explanatory variable (10 or fewer variables to explain variability in the outcome), allowing for 20% attrition [58–61]. Because we do not expect a multivariable model to exceed 10 predictors, the initial sample size estimation is unchanged. The definition of an asthma attack in both phases of the study is an episode of acute wheezing requiring an unscheduled visit to a health professional (including visits to ERs) and which is controlled by β_2-agonist bronchodilator treatment. The original definition for an asthma attack in Phase I that included ER attendance had to be modified in the light of changes in health seeking behaviour caused by the COVID-19 pandemic and fear of contagion in hospitals and other acute health facilities [19]. Potential explanatory variables include age, gender, ethnicity, socioeconomic status, medication use, asthma attacks and use of systemic corticosteroids in year prior to recruitment, asthma control test score, and study centre. Given changes to the Phase I protocol, an additional variable (for recruitment from ERs vs. hospital lists) will be considered as an effect modifier. Sub-group analyses of Phase II will include SARS-CoV-2 seropositivity as an explanatory variable. Secondary outcomes will include symptom severity and asthma control score.

Statistical analysis

The primary outcome will be the first asthma attack recurrence during 12 months of follow-up in Phase I and at least 6 months in Phase II. Both logistic regression and survival analyses will be used. For the latter, if no re-attendance related to an asthma attack occurred during the follow-up period, the patient is considered (right) censored. The general assumption for time-to-event (survival analysis) is that the censoring is non-informative, i.e. it is independent of the events (asthma attacks). Under this assumption, the results should not be qualitatively different from those inferred from a logistic regression. Odds ratios and/or hazard ratios will measure the associations between asthma attack recurrence and explanatory variables.

The Cox proportional hazard (PH) semi-parametric technique allows no assumption to be made on the baseline hazard; nevertheless, proportionality of the hazards should hold. The latter assumption can be tested on the basis of Shoenfeld residuals after

For peer review only - http://bmjopen.bmj.com/site/about/guidelines.xhtml

fitting models to the data. If unsatisfactory, alternative parametric models will be considered after understanding the distributional assumptions on the data such that the baseline hazard function is modelled appropriately.

After exploring the data as described above, a forward-backward elimination survival analysis would be applied to determine the most parsimonious model for an adjusted multivariable model; that is the model with the least number of predictors yet explaining most of the variability in the data. Penalized-likelihood criteria such as AIC or BIC will be used to choose between non-nested models – nevertheless based on complete observations only.

Hosmer-Lemeshow measure of goodness of fit would be employed for logistic regression models with a p-value of less than 0.05 indicating a poor fit to the data. Model validation will be done by splitting the data set into training-testing subsets (logistic regression setting). Cross validation techniques will be used to understand the prediction power of the corresponding logistic regression model using area under the receiver operator curve, prior to propose the model entering the clinical practice with the view of elaborating a prognostic model. In survival setting it is also possible that the precise time of the attacks may be missed but recorded as between two time points. This would fall into interval censored data as an alternative to survival setting in which the precise time of the event is required.

Multiple recurrences of asthma attacks within the follow-up period will generate a longitudinal binary outcome, with multiple records for each participant over follow-up. The hierarchical structure of the data requires special inferential techniques which account for the two sources of variability: that of between participants and within participants. Three different modelling approaches will be considered: generalized estimation equation, mixed models, and random effects survival models. The first approach provides population-average estimates, the second provides subject-specific estimates while the third is also known as shared frailty survival model. A frailty is a latent random effect which enters multiplicatively on the hazard function and accounts for the multiple failures setting and/or for the clustered aspect of the data.

Complete data analyses will be done under missing completely at random assumptions. However, patterns in missing data will be explored and methods to

For peer review only - http://bmjopen.bmj.com/site/about/guidelines.xhtml

evaluate potential impact of missing data on estimates will be used as appropriate such as multiple imputations using chained equations (MICE) accompanied by a sensitivity analysis to the missing data assumptions [62–64].

Analyses will use STATA (StataCorp. 2017. Stata Statistical Software: Release 15. College Station, TX: StataCorp LLC) or R (R Core Team (2013).

Discussion

The prevalence of childhood asthma has increased in many LMICs over the past 50 or so years where asthma attacks are a growing burden to health systems [2]. There are limited data on determinants of asthma attacks in children and adolescents from LMIC settings [18], and a need for prospective studies to identify risk factors for asthma attacks so that the limited resources available in low-resource settings can be focused on those children most likely to suffer recurrent attacks to reduce morbidity and economic costs to health systems and patients' families. The Asthma ATTACK study aims to identify risk factors associated with asthma attacks among children and adolescents attending emergency rooms in public hospitals in three Ecuadorian cities.

This study provides a range of climatic settings in Ecuador from high altitude (Cuenca and Quito) to sea level (Portoviejo). Study activities were interrupted in March 2020 by the Covid-19 pandemic resulting in a suspension of recruitment in hospital ERs owing to dramatic changes in access to and use of health facilities. As a consequence, the study protocol was modified to allow re-initiation of recruitment and also the evaluation of the effect of exposures to SARS-CoV-2 on risk of asthma attacks and symptoms in children. The study will help fill an unmet knowledge gap on the effects of SARS-CoV-2 on pediatric asthma, particularly from low-resource LMIC settings [19].

Ethics and dissemination

Ethical approval for Phase I was obtained from the Ethics Committee of the Hospital General Docente de Calderon (CEISH-HGDC 2019-001) and for Phase II from the Ecuadorian Ministry of Public Health COVID-19 Ethics Committee (MSP-CGDES-2021-0041-O N° 096-2021).

For peer review only - http://bmjopen.bmj.com/site/about/guidelines.xhtml

References

1. Reddel HK, Busse WW, Pedersen S, Tan WC, Chen Y-Z, Jorup C, et al. Should recommendations about starting inhaled corticosteroid treatment for mild asthma be based on symptom frequency: a post-hoc efficacy analysis of the START study. Lancet. 2017;389: 157–166.

2. Pearce N, Aït-Khaled N, Beasley R, Mallol J, Keil U, Mitchell E, et al. Worldwide trends in the prevalence of asthma symptoms: phase III of the International Study of Asthma and Allergies in Childhood (ISAAC). Thorax. 2007;62: 758–766.

3. Hasegawa K, Bittner JC, Nonas SA, Stoll SJ, Watase T, Gabriel S, et al. Children and Adults With Frequent Hospitalizations for Asthma Exacerbation, 2012-2013: A Multicenter Observational Study. J Allergy Clin Immunol Pract. 2015;3: 751–8.e1.

4. Deschildre A, Pichavant M, Engelmann I, Langlois C, Drumez E, Pouessel G, et al. Virus-triggered exacerbation in allergic asthmatic children: neutrophilic airway inflammation and alteration of virus sensors characterize a subgroup of patients. Respir Res. 2017;18: 191.

5. Murray CS, Foden P, Sumner H, Shepley E, Custovic A, Simpson A. Preventing Severe Asthma Exacerbations in Children. A Randomized Trial of Mite-Impermeable Bedcovers. Am J Respir Crit Care Med. 2017;196: 150–158.

6. Taquechel K, Diwadkar AR, Sayed S, Dudley JW, Grundmeier RW, Kenyon CC, et al. Pediatric Asthma Health Care Utilization, Viral Testing, and Air Pollution Changes During the COVID-19 Pandemic. J Allergy Clin Immunol Pract. 2020;8: 3378–3387.e11.

7. Bønnelykke K, Sleiman P, Nielsen K, Kreiner-Møller E, Mercader JM, Belgrave D, et al. A genome-wide association study identifies CDHR3 as a susceptibility locus for early childhood asthma with severe exacerbations. Nat Genet. 2014;46: 51–55.

8. Fischer GB, Sarria EE, Camargos P, Mocelin HT, Soto-Quiroz M, Cruz AA, et al. Childhood asthma in low and middle-income countries: Where are we now? Paediatr Respir Rev. 2019;31: 52–57.

9. Carroll WD, Wildhaber J, Brand PLP. Parent misperception of control in childhood/adolescent asthma: the Room to Breathe survey. Eur Respir J. 2012;39: 90–96.

10. Vervloet M, van Dijk L, Spreeuwenberg P, Price D, Chisholm A, Van Ganse E, et al. The Relationship Between Real-World Inhaled Corticosteroid Adherence and Asthma Outcomes: A Multilevel Approach. J Allergy Clin Immunol Pract. 2020;8:

For peer review only - http://bmjopen.bmj.com/site/about/guidelines.xhtml

626–634.

11. Fleming L. Asthma exacerbation prediction: recent insights. Curr Opin Allergy Clin Immunol. 2018;18: 117–123.

12. Nunes C, Pereira AM, Morais-Almeida M. Asthma costs and social impact. Asthma Res Pract. 2017;3: 1.

13. Ardura-Garcia C, Arias E, Hurtado P, Bonnett LJ, Sandoval C, Maldonado A, et al. Predictors of severe asthma attack re-attendance in Ecuadorian children: a cohort study. Eur Respir J. 2019;54. doi:10.1183/13993003.02419-2018

14. Smiley M, Sicignano N, Rush T, Lee R, Allen E. Outcomes of follow-up care after an emergency department visit among pediatric asthmatics in the military health system. J Asthma. 2016;53: 816–824.

15. Beck AF, Simmons JM, Huang B, Kahn RS. Geomedicine: area-based socioeconomic measures for assessing risk of hospital reutilization among children admitted for asthma. Am J Public Health. 2012;102: 2308–2314.

16. Zipkin R, Schrager SM, Nguyen E, Mamey MR, Banuelos I, Wu S. Association between pediatric home management plan of care compliance and asthma readmission. J Asthma. 2017;54: 761–767.

17. Moncrief T, Beck AF, Simmons JM, Huang B, Kahn RS. Single parent households and increased child asthma morbidity. J Asthma. 2014;51: 260–266.

18. Ardura-Garcia C, Stolbrink M, Zaidi S, Cooper PJ, Blakey JD. Predictors of repeated acute hospital attendance for asthma in children: A systematic review and meta-analysis. Pediatr Pulmonol. 2018;53: 1179–1192.

19. Ochoa-Avilés AM, Ochoa-Avilés C, Morillo-Argudo DA, Molina-Cando MJ, Rodas-Espinoza CR, Chis Ster I, et al. Impact of COVID-19 pandemic on asthma symptoms and management: A prospective analysis of asthmatic children in Ecuador. World Allergy Organ J. 2021;14: 100551.

20. McGeachie MJ, Yates KP, Zhou X, Guo F, Sternberg AL, Van Natta ML, et al. Patterns of Growth and Decline in Lung Function in Persistent Childhood Asthma. N Engl J Med. 2016;374: 1842–1852.

21. Forno E, Gogna M, Cepeda A, Yañez A, Solé D, Cooper P, et al. Asthma in Latin America. Thorax. 2015;70: 898–905.

22. Alith MB, Gazzotti MR, Nascimento OA, Jardim JR. Impact of asthma control on different age groups in five Latin American countries. World Allergy Organ J. 2020;13: 100113.

23. Gold LS, Thompson P, Salvi S, Faruqi RA, Sullivan SD. Level of asthma control and

For peer review only - http://bmjopen.bmj.com/site/about/guidelines.xhtml

health care utilization in Asia-Pacific countries. Respir Med. 2014;108: 271–277.

24. Sastre J, Fabbri LM, Price D, Wahn HU, Bousquet J, Fish JE, et al. Insights, attitudes, and perceptions about asthma and its treatment: a multinational survey of patients from Europe and Canada. World Allergy Organ J. 2016;9: 13.

25. Reddel HK, Sawyer SM, Everett PW, Flood PV, Peters MJ. Asthma control in Australia: a cross-sectional web-based survey in a nationally representative population. Medical Journal of Australia. 2015. pp. 492–496. doi:10.5694/mja14.01564

26. Ardura-Garcia C, Vaca M, Oviedo G, Sandoval C, Workman L, Schuyler AJ, et al. Risk factors for acute asthma in tropical America: a case-control study in the City of Esmeraldas, Ecuador. Pediatric Allergy and Immunology. 2015. pp. 423–430. doi:10.1111/pai.12401

27. Cruz AA, Souza-Machado A, Franco R, Souza-Machado C, Ponte EV, Santos PM, et al. The Impact of a Program for Control of Asthma in a Low-Income Setting. World Allergy Organization Journal. 2010. pp. 167–174. doi:10.1097/wox.0b013e3181dc3383

28. Cusack RP, Satia I, O'Byrne PM. Asthma maintenance and reliever therapy: Should this be the standard of care? Ann Allergy Asthma Immunol. 2020;125: 150–155.

29. Abrams EM, Becker AB, Szefler SJ. Paradigm Shift in Asthma Therapy for Adolescents: Should It Apply to Younger Children as Well? JAMA pediatrics. 2020. pp. 227–228.

30. Abrams EM, Shaker M, Greenhawt M, Fernandes RM, Sinha I. Treatment of mild-to-moderate asthma in childhood and adolescence in 2021. Lancet Respir Med. 2021;9: 443–445.

31. Reddel HK, FitzGerald JM, Bateman ED, Bacharier LB, Becker A, Brusselle G, et al. GINA 2019: a fundamental change in asthma management: Treatment of asthma with short-acting bronchodilators alone is no longer recommended for adults and adolescents. Eur Respir J. 2019;53. doi:10.1183/13993003.01046-2019

32. Neffen H, Chahuàn M, Hernández DD, Vallejo-Perez E, Bolivar F, Sánchez MH, et al. Key factors associated with uncontrolled asthma – the Asthma Control in Latin America Study. Journal of Asthma. 2020. pp. 113–122. doi:10.1080/02770903.2018.1553050

33. Liu S, Zhi Y, Ying S. COVID-19 and Asthma: Reflection During the Pandemic. Clin Rev Allergy Immunol. 2020;59: 78–88.

34. Wang Y, Ao G, Qi X, Xie B. The association between COVID-19 and asthma: A

For peer review only - http://bmjopen.bmj.com/site/about/guidelines.xhtml

systematic review and meta-analysis. Clinical & Experimental Allergy. 2020. pp. 1274–1277. doi:10.1111/cea.13733

35. Hartmann-Boyce J, Gunnell J, Drake J, Otunla A, Suklan J, Schofield E, et al. Asthma and COVID-19: review of evidence on risks and management considerations. BMJ Evid Based Med. 2020. doi:10.1136/bmjebm-2020-111506

36. Peters MC, Sajuthi S, Deford P, Christenson S, Rios CL, Montgomery MT, et al. COVID-19-related Genes in Sputum Cells in Asthma. Relationship to Demographic Features and Corticosteroids. Am J Respir Crit Care Med. 2020;202: 83–90.

37. Yamaya M, Nishimura H, Deng X, Sugawara M, Watanabe O, Nomura K, et al. Inhibitory effects of glycopyrronium, formoterol, and budesonide on coronavirus HCoV-229E replication and cytokine production by primary cultures of human nasal and tracheal epithelial cells. Respir Investig. 2020;58: 155–168.

38. Schultze A, Douglas I. COVID-19 and inhaled corticosteroids—another piece in an expanding puzzle. The Lancet Respiratory Medicine. 2021. pp. 674–675. doi:10.1016/s2213-2600(21)00076-x

39. Papadopoulos NG, Mathioudakis AG, Custovic A, Deschildre A, Phipatanakul W, Wong G, et al. Childhood asthma outcomes during the COVID-19 pandemic: Findings from the PeARL multi-national cohort. Allergy. 2021;76: 1765–1775.

40. Kaye L, Theye B, Smeenk I, Gondalia R, Barrett MA, Stempel DA. Changes in medication adherence among patients with asthma and COPD during the COVID-19 pandemic. J Allergy Clin Immunol Pract. 2020;8: 2384–2385.

41. Instituto Nacional de Meteorología e Hidrología. Anuario Meteorológico Nro. 53-2013. Quito, Ecuador: INAMHI; 2015.

42. Ministerio de Salud Pública. Manual del modelo de atención integral del sistema nacional de salud familiar comunitario e intercultural (MAIS-FCI). Ecuador: MAIS-FCI; 2012 [cited 22 Apr 2021]. Available: https://es.scribd.com/document/338645477/Manual-MAIS-MSP12-12-12-pdf

43. Lucio R, Villacrés N, Henríquez R. Sistema de salud de Ecuador. Salud pública de México. 2011;53: s177–s187.

44. Liu AH, Zeiger R, Sorkness C, Mahr T, Ostrom N, Burgess S, et al. Development and cross-sectional validation of the Childhood Asthma Control Test. J Allergy Clin Immunol. 2007;119: 817–825.

45. Nathan RA, Sorkness CA, Kosinski M, Schatz M, Li JT, Marcus P, et al. Development of the asthma control test☆A survey for assessing asthma control. Journal of Allergy and Clinical Immunology. 2004. pp. 59–65. doi:10.1016/j.jaci.2003.09.008

For peer review only - http://bmjopen.bmj.com/site/about/guidelines.xhtml

46. Juniper EF, Guyatt GH, Feeny DH, Ferrie PJ, Griffith LE, Townsend M. Measuring quality of life in children with asthma. Qual Life Res. 1996;5: 35–46.

47. Fitzclarence CA, Henry RL. Validation of an asthma knowledge questionnaire. J Paediatr Child Health. 1990;26: 200–204.

48. Praena Crespo M, Lora Espinosa A, Aquino Llinares N, Sánchez Sánchez AM, Jiménez Cortés A. [The Spanish version of the Newcastle Asthma Knowledge Questionnaire for parents of children with asthma (NAKQ). Transcultural adaptation and reliability analysis]. An Pediatr . 2009;70: 209–217.

49. Franco R, Nascimento HF, Cruz AA, Santos AC, Souza-Machado C, Ponte EV, et al. The economic impact of severe asthma to low-income families. Allergy. 2009;64: 478–483.

50. Quanjer PH, Stanojevic S, Cole TJ, Baur X, Hall GL, Culver BH, et al. Multi-ethnic reference values for spirometry for the 3-95-yr age range: the global lung function 2012 equations. Eur Respir J. 2012;40: 1324–1343.

51. Association for Respiratory Technology & Physiology. ARTP COVID-19 GROUP: Practical guidance for increasing Lung Function and Sleep activity during COVID-19 Endemic Phase. Aug 2020 [cited 25 Jan 2021]. Available: https://www.artp.org.uk/write/MediaUploads/Standards/COVID19/ARTP_COVID_Practical_Innovations_Vers1.2_final.pdf

52. PCRS. Position Statement Diagnostic work up of the patient presenting with respiratory symptoms during the COVID-19 pandemic. In: The Primary Care Respiratory Society [Internet]. 17 Sep 2020 [cited 25 Jan 2021]. Available: https://www.pcrs-uk.org/sites/pcrs-uk.org/files/resources/COVID19/Diagnosis-asthma-COPD-during-Covid-19-v2-17-September-2020.pdf

53. Coronavirus & the 2nd Generation NObreath - Update. In: NObreath [Internet]. [cited 25 Jan 2021]. Available: https://www.nobreathfeno.com/coronavirus-and-the-2nd-generation-nobreath-update/

54. Guevara Á, Vivero S, Nipaz V, Guaraca V, Coloma J. A Low-Cost SARS-CoV-2 rRBD ELISA to Detect Serostatus in Ecuadorian Population with COVID-19. Am J Trop Med Hyg. 2021. doi:10.4269/ajtmh.20-1420

55. AAAAI. Spirometry during COVID-19. In: American Academy of Allergy, Asthma & Immunology [Internet]. 23 Apr 2020 [cited 25 Jan 2021]. Available: https://www.aaaai.org/ask-the-expert/spirometry

56. Stanojevic S, Beaucage F, Comondore V, Faughnan M, Kovesi T, McCoy C, et al. Resumption of pulmonary function testing during the post-peak phase of the COVID-19 pandemic. Canadian Journal of Respiratory, Critical Care, and Sleep Medicine. 2020. pp. 156–159. doi:10.1080/24745332.2020.1796211

For peer review only - http://bmjopen.bmj.com/site/about/guidelines.xhtml

57. McGowan A, Sylvester K, Burgos F, Boros P, de Jongh F, Kendrick A, et al. Recommendation from ERS Group 9.1 (Respiratory function technologists/Scientists) Lung function testing during COVID-19 pandemic and beyond. ; 2020 [accessed 2020 Aug 31]. In: European Respiratory Society [Internet]. [cited 18 Jul 2020]. Available: https://ers.app.box.com/s/zs1uu88wy51monr0ewd990itoz4tsn2h

58. Vittinghoff E, McCulloch CE. Relaxing the rule of ten events per variable in logistic and Cox regression. Am J Epidemiol. 2007;165: 710–718.

59. Peduzzi P, Concato J, Kemper E, Holford TR, Feinstein AR. A simulation study of the number of events per variable in logistic regression analysis. J Clin Epidemiol. 1996;49: 1373–1379.

60. Concato J, Peduzzi P, Holford TR, Feinstein AR. Importance of events per independent variable in proportional hazards analysis. I. Background, goals, and general strategy. J Clin Epidemiol. 1995;48: 1495–1501.

61. Peduzzi P, Concato J, Feinstein AR, Holford TR. Importance of events per independent variable in proportional hazards regression analysis. II. Accuracy and precision of regression estimates. J Clin Epidemiol. 1995;48: 1503–1510.

62. White IR, Royston P, Wood AM. Multiple imputation using chained equations: Issues and guidance for practice. Stat Med. 2011;30: 377–399.

63. White IR, Royston P. Imputing missing covariate values for the Cox model. Stat Med. 2009;28: 1982–1998.

64. Royston P, Altman DG. External validation of a Cox prognostic model: principles and methods. BMC Med Res Methodol. 2013;13: 33.

Author statement: Study design – PJC, AAC, NR, MB, MP, CA-G; study conduct – DM, SM-B, AO, MEC, CR, AM, KA, AR, CF; manuscript drafting – PJC, DM, SM-B; statistical analysis plan – ICS; manuscript editing and review – all authors.

Funding: This work is based on the Asthma Attacks Causes and Prevention Study in Urban Latin America which is funded by the National Institute of Health Research [NIHR] under the Global Health Research Programme [grant 17/63/62]. Additional

financial support was provided by the Corporación Ecuatoriana para el Desarrollo de la Investigación y la Academia CEDIA [N° CEPRA-XV-2021-03].

Competing Interests statement: The authors declare no competing interests.

Patient and public involvement statement: There was no patient or public involvement in the design or conduct of this study. Results of tests are being provided at the time of testing and the study population is being informed of study results through periodic leaflets, a website, social media texts and videos, and study group online meetings.

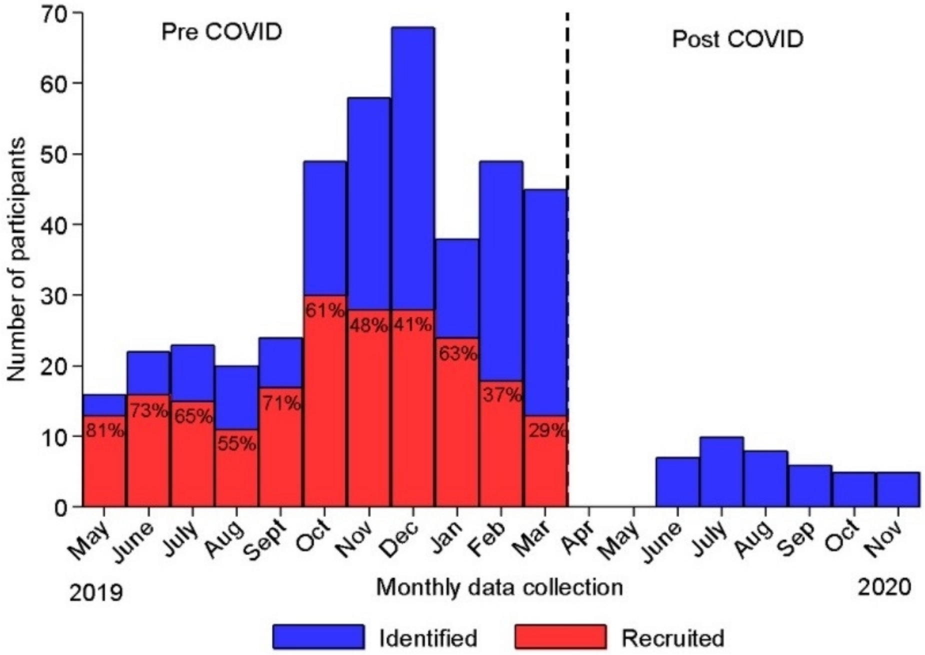

Figure 1

152x107mm (144 x 144 DPI)

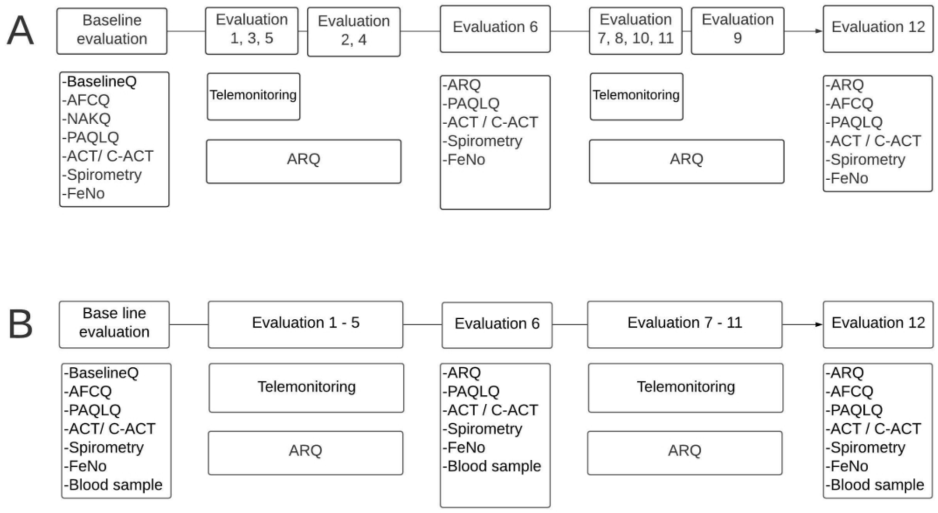

Figure 2

408x217mm (160 x 160 DPI)

BMJ Open

A prospective study of factors associated with asthma attack recurrence (ATTACK) in children from three Ecuadorian cities during COVID-19: a study protocol

Journal:	*BMJ Open*
Manuscript ID	bmjopen-2021-056295.R1
Article Type:	Protocol
Date Submitted by the Author:	28-Apr-2022
Complete List of Authors:	Morillo, Diana; Universidad Internacional del Ecuador<br>Mena-Bucheli, Santiago; Universidad Internacional del Ecuador<br>Ochoa, Angélica; Universidad de Cuenca<br>Chico, Martha E; Universidad Internacional del Ecuador<br>Rodas, Claudia; Universidad de Azuay<br>Maldonado, Augusto; Universidad San Francisco de Quito; Hospital General Docente Calderón<br>Arteaga, Karen; Hospital Verdi Cevallos Balda<br>Alchundia, Jessica; Hospital de Especialidades Portoviejo<br>Solorzano, Karla; Hospital de Especialidades Portoviejo<br>Rodriguez, Alejandro ; Universidad Internacional del Ecuador<br>Figueiredo, Camila; Universidade Federal da Bahia<br>Ardura-Garcia, Cristina; University of Bern<br>Bachmann, Max; Univeristy of East Anglia, Norwich Medical School, University of East Anglia<br>Perkin, Michael; St George's, Population Health Research Institute<br>Chis Ster, Irina; St. George's University of London; St George's University of London<br>Cruz, Alvaro; Universidade Federal da Bahia<br>Romero, Natalia; Universidad Internacional del Ecuador, Quito, School of Medicine; Grups de Recerca d'America i Africa Llatines, GRAAL<br>Cooper, Philip; Universidad Internacional del Ecuador; St George’s University of London
**Primary Subject Heading**:	Respiratory medicine
Secondary Subject Heading:	Global health, Epidemiology, Public health
Keywords:	Asthma < THORACIC MEDICINE, COVID-19, Epidemiology < THORACIC MEDICINE

SCHOLARONE™
Manuscripts

Title: A prospective study of factors associated with asthma attack recurrence (ATTACK) in children from three Ecuadorian cities during COVID-19: a study protocol

Authors

Diana Morillo[1], Santiago Mena-Bucheli[1], Angélica Ochoa[2], Martha E Chico[1], Claudia Rodas[3], Augusto Maldonado [4,5], Karen Arteaga[6], Jessica Alchundia[7], Karla Solorzano[7], Alejandro Rodriguez[1], Camila Figueiredo[8], Cristina Ardura-Garcia[9], Max Bachmann[10], Michael Perkin[11], Irina Chis Ster[11], Alvaro A Cruz[8, 12], *Natalia Romero[1,13], *Philip J Cooper [1,11]

Affiliations

[1] Universidad Internacional del Ecuador, Quito, Ecuador.

[2] Universidad de Cuenca, Cuenca, Ecuador.

[3] Universidad de Azuay, Cuenca, Ecuador

[4] Universidad San Francisco de Quito, Quito, Ecuador

[5] Hospital General Docente Calderón, Quito, Ecuador

[6] Hospital Verdi Cevallos Balda, Portoviejo, Ecuador

[7] Hospital de Especialidades, Portoviejo, Ecuador

[8] Universidade Federal da Bahia, Salvador, Brazil

[9]Institute of Social and Preventive Medicine, University of Bern, Bern, Switzerland.

[10] University of East Anglia, Norwich, UK.

[11] St George's University of London, London, UK.

[12]ProAR-Center of Excellence in Asthma, Salvador, Brazil

[13]Grups de Recerca d'Amèrica i Àfrica Llatines GRAAL, Barcelona, Spain

*Contributed equally

Correspondence

Philip J. Cooper, Escuela de Medicina, Universidad Internacional del Ecuador, Av. Jorge Fernández y Av. Simon Bolivar, Quito, Ecuador.

Email: pcooper@sgul.ac.uk

For peer review only - http://bmjopen.bmj.com/site/about/guidelines.xhtml

Word count
Abstract: 299
Text: 4,372

For peer review only - http://bmjopen.bmj.com/site/about/guidelines.xhtml

Strengths and limitations of this study

- Asthma attacks are a growing cause of morbidity in children and burden on health systems in low and middle-income countries, and there is limited information on factors associated with asthma attacks in poor urban settings in Latin America.
- This protocol describes a study designed to investigate risk factors associated with recurrence of asthma attacks in children and adolescents living in marginalized settings in three Ecuadorian cities.
- This study is recruiting children and adolescents with uncontrolled asthma symptoms and asthma attacks and following them prospectively for 6 to 12 months to identify factors associated with recurrence of attacks in the context of the COVID-19 pandemic.
- The COVID-19 pandemic has had a major impact on health seeking behaviours while mitigation strategies to control transmission of the novel coronavirus, SARS-CoV-2, have resulted in marked declines in the circulation of respiratory viruses that are considered to underlie a high proportion of asthma attacks. Such factors may interfere with recruitment and follow-up of study participants.

For peer review only - http://bmjopen.bmj.com/site/about/guidelines.xhtml

Abstract

Introduction: Asthma is a growing health problem in children in marginalized urban settings in low and middle-income countries. Asthma attacks are an important cause of emergency care attendance and long-term morbidity. We designed a prospective study, the Asthma Attacks study (ATTACK), to identify factors associated with recurrence of asthma attacks (or exacerbations) among children and adolescents attending emergency care in three Ecuadorian cities.

Methods and analysis: Prospective cohort study designed to identify risk factors associated with recurrence of asthma attacks in 450 children and adolescents aged 5 to 17 years attending emergency care in public hospitals in three Ecuadorian cities (Quito, Cuenca, and Portoviejo). The primary outcome will be rate of asthma attack recurrence during up to 12 months of follow-up. Data are being collected at baseline and during follow-up by questionnaire: sociodemographic data, asthma history and management (baseline only); recurrence of asthma symptoms and attacks (monthly); economic costs of asthma to family; Asthma Control Test; Pediatric Asthma Quality of life Questionnaire; and Newcastle Asthma Knowledge Questionnaire (baseline only). In addition, the following are being measured at baseline and during follow-up: lung function and reversibility by spirometry before and after salbutamol; fractional exhaled nitric oxide (FeNO); and presence of IgG antibodies to SARS-CoV-2 in blood. Recruitment started in 2019 but because of severe disruption to emergency services caused by the COVID-19 pandemic, eligibility criteria were modified to include asthmatic children with uncontrolled symptoms and registered with collaborating hospitals. Data will be analysed using logistic regression and survival analyses.

Ethics and dissemination: Ethical approval was obtained from the Hospital General Docente de Calderon (CEISH-HGDC 2019-001) and Ecuadorian Ministry of Public Health (MSP-CGDES-2021-0041-O N° 096-2021). The study results will be disseminated through presentations at conferences and to key stakeholder groups including policy makers, postgraduate theses, peer-review publications, and a study website.

For peer review only - http://bmjopen.bmj.com/site/about/guidelines.xhtml

Introduction

Asthma is the most common chronic disease of childhood and is estimated to affect more than 350 million people worldwide [1]. Although initially described as a disease of high-income countries (HICs) such as the United Kingdom, international comparisons using standardized methodologies have shown a similar or greater prevalence among children in urban centres in Latin America as observed in HICs [2]. There is evidence that the prevalence of asthma has increased in low- and middle-income countries (LMICs) over recent decades [2].

Asthma attacks or exacerbations are frequent in children and are a common cause of hospitalization [3], most frequently associated with respiratory viral infections [4], but also with exposures to allergens [5] and air pollution [6] among individuals with an underlying genetic susceptibility [7]. Asthma is an underdiagnosed disease because of lack of specialized personnel and resources to evaluate lung function. Further, limited access to specialized care and treatment [8], accompanied by poor treatment adherence [9,10] are important contributing factors to inadequate disease control and attacks, especially in underprivileged populations. In such settings, asthma patients with poor control of their daily symptoms often rely on emergency room care for management of their symptoms [11], resulting in high economic costs to health systems and patients' families [8,12].

To understand better factors contributing to the risk of asthma attacks, it is important identify those factors contributing to suboptimal management which can be improved through better health care [13]. Several studies have investigated potential predictors of recurrence of asthma attacks among children attending emergency services. Factors identified as important included a history of previous emergency attendances for acute asthma attacks, younger age [13–15], ethnicity of African descent [16,17], and low socioeconomic status [13,18]. These studies were conducted almost exclusively in North America and frequently included adult Hispanic populations living there. There are relatively few such studies done in LMICs [13,16,18].

The Asthma Attacks Study (ATTACK) aims to identify factors related to recurrence of asthma attacks in Ecuadorian children attending emergency care in three Ecuadorian cities. Recruitment into the study started in March 2019 but was interrupted by the COVID-19 pandemic that had a major impact on use of and access to emergency care [19]. In consequence, the original protocol was modified to allow continued recruitment and measure the impact of the COVID-19 pandemic and exposures to SARS-CoV-2 on asthma attacks in children and adolescents.

What is known about factors related to asthma attacks and impact of COVID-19 pandemic?

Severe asthma in children is associated with loss of lung function [20], as well as with high costs of medical and family care [2]. In Latin America, asthma has been described as a major public health problem because of a high prevalence and significant associated morbidity and mortality [21]. Asthma control among children in Latin America has been reported to be among the worse worldwide [22–25]. The Latin America Asthma Insights and Management study reported adequate asthma control in less than 20% of asthmatic children in Argentina, Brazil, Mexico, Venezuela, and Puerto Rico [22]. A case-control study of asthmatic children with an acute attack attending an Emergency Room (ER) in coastal Ecuador showed that, over the previous 12 months, only 20% of children had visited a doctor in the past year for a routine visit, 86% had attended an emergency room at least once prior, and none were receiving inhaled corticosteroids despite over half having suffered four or more acute exacerbations [26]. A previous prospective study of children with asthma attacks in coastal Ecuador showed a recurrence rate of 46% within 6 months [13].

Asthma attacks represent an acute or subacute increase in respiratory symptoms [25] and severe attacks - defined as requiring ER or hospital care and treatment with systemic corticosteroids for at least 3 days - are associated with loss of lung function [20] and high economic costs to the patient’s family and health care system [7], as well as absenteeism in work and school.

Asthma attacks may be prevented either by avoiding previously identified triggers or by appropriate preventive treatment. Patients using inhaled corticosteroids (ICS) were 3 times more likely to have well-controlled asthma [22], a reduction in symptoms independent of frequency, and better lung function [1]. In Brazil, preventive strategies have been successfully implemented through asthma control programs such as Pro-AR in Salvador de Bahía to reduce future risk in asthmatic patients from less privileged backgrounds, through the provision of ICS [27]. There has been a shift to using ICS more widely for control of asthma, for example, through the provision of combination inhalers including both ICS and a beta$_2$ agonist for moderate to severe asthma [28]. More recent evidence indicate that the use of ICS may be beneficial even for asthmatic children with mild disease that accounts for 30-40% of severe attacks [29]. Treatment recommendations for children and adolescents with mild disease are now shifting to use of ICS (plus long-acting beta-agonists in combination inhalers) as required [30,31]. Despite such initiatives, access to ICS in many LMIC settings is limited within health care systems because of cost and a failure to recognize the public health impact of childhood asthma. In such settings, children with asthma symptoms frequently over-rely on ER care for the acute control of their symptoms in the absence of adequate provision of specialized care for their long-term management [26,32].

Soon after the emergence of the novel coronavirus, SARS-CoV-2, in humans and its spread into a global pandemic, the question was raised as to whether this novel viral infection might alter the risk of asthma symptoms because of deficient antiviral immune responses and the tendency to increase exacerbations during infections with other respiratory viruses [33]. To date, evidence from studies of asthmatic children and adults do not indicate an increased risk [34,35]. Characteristics of asthmatic airways among those with T2-linked phenotypes may reduce the expression of the cellular receptor for SARS-CoV-2, angiotensin-converting enzyme 2 (ACE2), in the airways [36]. It has been suggested that the use of ICS among asthmatics may reduce risk of severe COVID-19 by suppressing inflammation and enhancing antiviral defenses [37], although the evidence remains unclear [38]. There are limited data from pediatric populations [38]. The COVID-19 pandemic has resulted in marked reductions in

hospital visits including to emergency rooms (Figure 1), reduced attack rates, and better lung function among children with asthma [19,39], which are likely consequences of mitigation measures to reduce transmission of SARS-CoV-2 (such as lockdowns, face masks, and social distancing) that have resulted also in the reduced transmission of other respiratory viruses [4,6,10,39]. Treatment adherence may have improved also because of better parental supervision of asthma medications [40]. There are limited data on the impact of exposure to SARS-CoV-2 on asthma attacks and uncontrolled symptoms and the impact of the collateral effects of the COVID-19 pandemic including reduced access to health services and medications and effects of COVID-19 mitigation efforts in resource-poor LMIC settings.

Figure 1: Impact of the COVID-19 pandemic in the 3 study centres in Quito, Portoviejo and Cuenca, Ecuador, on recruitment rates into the study and number of potentially eligible subjects identified in collaborating emergency rooms. Red bars show numbers recruited as a proportion (%) of those eligible (blue bars) prior to a legally enforced national lockdown on 16th March 2020.

Methods and analysis

The Asthma Attack study

A prospective multi-center study is being done in children aged 5 to 17 years in low-income settings in three Ecuadorian cities (Quito, Cuenca, and Portoviejo) to study factors associated with acute asthma attacks. Because of the impact of the COVID-19 pandemic on risk of asthma attacks and health care access, the study was divided into 2 phases of recruitment and follow-up: 1) Phase I (or pre-COVID-19 from May 2019 to March 2020) evaluated risk factors associated with recurrence of acute asthma attacks among children and adolescents attending emergency rooms of public hospitals in low-income settings in the three cities; and 2) Phase II (or peri-COVID-19 from March 2021 to June 2022) is evaluating risk factors associated with attacks among children and adolescents with asthma and current symptoms who have previously attended specialist care at public hospitals in Cuenca and Portoviejo. Phase II is also evaluating the effects of exposures to SARS-CoV-2 on asthma attacks and symptoms. In addition,

both study phases are evaluating the impact of asthma symptoms and adequate control of asthma on the quality of life and economic costs for the family of children and adolescents with asthma. The objectives of Phases I and II are detailed in Table 1. Qualitative studies of health worker's perspectives on asthma care coordination between primary and specialized health care are being done also as described elsewhere [41].

Study design and setting

This is a multi-center prospective study conducted in three Ecuadorian cities (Quito, Cuenca, and Portoviejo). Quito, the capital, and Cuenca, the third largest city, are located in the Andean highlands (altitude >2,500m) where the annual average temperature is ~16°C, while Portoviejo is in the coastal region (at sea level) with an annual average temperature of ~25 °C [42]. According to the 2010 census, the populations of the three cities are: Quito >2 million, Cuenca 0.5 million, and Portoviejo 0.28 million inhabitants. Ecuador is an upper-middle-income-country with a per capita income of $6,080 in 2019. The health system in Ecuador includes public and private provision. Public institutions offer universal health coverage that is stratified into different levels of care from primary to tertiary services [43]. Social security institutions offer health services to affiliated salaried workers and their families. The private sector includes for-profit entities (hospitals, clinics, dispensaries, doctor's offices, pharmacies, and prepaid health insurance companies) which are generally located in the main cities [44].

Study centers

The study was initially based in the tertiary care hospitals in each of the three cities (Hospital Docente General de Calderon, Quito; Hospital Vicente Corral Moscoso, Cuenca; and Hospital Verdi Cevallos Balda, Portoviejo).

PHASE	OBJECTIVES	HOW OBJECTIVE WILL BE ACHIEVED	STATISTICAL OUTCOME	STATISTICAL METHODS	STATISTICAL INFERENCE
**1**	**PRIMARY:**				
	Risk factors associated with asthma attacks recurrence requiring an emergency visit during follow-up	Monthly follow-up and recording of events	Binary: Yes/No indicating at least one individual recurrence	Logistic regression	OR and 95% CIs;<br>Predictive model for recurrence, ROC and AUC
	**SECONDARY:**				
	1. Risk factors associated with time to first asthma attack recurrence<br><br>2. Risk factors associated with monthly asthma attack recurrence<br><br>3. Evaluate impact of asthma recurrence and control on quality of life and economic costs for patient's families.	Monthly follow-up and recording of events<br><br>Monthly follow-up and recording of events<br><br>Monthly follow-up and recording of events and questionnaire on asthma control (0, 6, & 12 months) and quality of life and economic costs at 6 & 12 months.	Binary: Yes/No indicating the first recurrence and/or time to the first event<br>Longitudinal binary outcomes indicating events and times<br><br>Longitudinal continuous outcomes indicating scores for quality of life and economic costs	Survival analyses modelling time to first event<br>Longitudinal binary outcome<br><br>Longitudinal continuous data outcomes and time-varying covariates	HR and 95% CIs; Survival models – proportional hazard t (semi-parametric Cox or parametric Weibull) or accelera time failure depending on data<br>OR; HR and 95 %CIs;<br>Longitudinal binary models (GEE)<br>Multiple events per individual - frailty survival analysis<br>Longitudinal continuous data analysis with time varying binary covariates indicating presence of recurrent event
**2**	**PRIMARY:**				
	Risk factors associated with asthma attacks recurrence requiring an emergency visit during follow-up	Monthly follow-up and recording of events	Binary: Yes/No indicating at least one individual recurrence during follow-up	Logistic regression	OR and 95% CIs;<br>Predictive model, ROC, AUC
	**SECONDARY:**				
	1. Risk factors associated with time to first asthma attack recurrence<br><br>2. Risk factors associated with monthly asthma attack recurrence<br><br>3. Evaluate impact of asthma recurrence and control on quality of life and economic costs for patient's families.<br><br>4. Effects of seropositivity to SARS-CoV-2 on risk of any asthma attack recurrence and number of events	Monthly follow-up and recording of events<br><br>Monthly follow-up and recording of events<br><br>Monthly follow-up and recording of events and questionnaire on asthma control (0, 6, & 12 months) and quality of life and economic costs at 6 & 12 months.<br>Serology for SARS-CoV-2 at 0 & 6 months	Binary: Yes/No indicating the first recurrence and/or time to first event<br>Longitudinal binary outcomes indicating events and times<br><br>Longitudinal continuous outcomes indicating scores for quality of life and economic costs<br><br>Longitudinal binary outcomes indicating events and times	Survival type analyses modelling time to first event<br>Longitudinal binary outcome<br><br>Longitudinal continuous outcomes and time-varying covariates<br><br>Longitudinal binary outcome and time-varying covariates	HR and 95% CIs; Survival models – proportional hazard t (semi-parametric Cox or parametric Weibull) or accelera time failure depending on data<br>OR; HR and 95% CIs;<br>Longitudinal binary models (GEE estimation)<br>Multiple events per individual - frailty survival analysis<br>OR and 95% CIs;<br>Longitudinal continuous data analysis with time varying binary covariates indicating presence of recurrent event<br><br>Longitudinal binary analysis with time varying binary indicating SARS-CoV-2 serology. Both population averag (PA) and subject-specific (SS) models to be considered g time-varying nature of the SARS-CoV-2 variable

For peer review only - http://bmjopen.bmj.com/site/about/guidelines.xhtml

Table 1. Study objectives for Phases I and II, how each objective will be achieved, and statistical analysis for each objective

OR - ODDS RATIOS; 95% CIs – 95% confidence intervals; HR – Hazard ratios; ROC- Receiving operator characteristic; AUC - area under the curve; GEE – generalized estimating equations

For peer review only - http://bmjopen.bmj.com/site/about/guidelines.xhtml

Recruitment - Phase I (pre-COVID-19; May 2019 to March 2020)

Children and adolescents aged 5–17 years were recruited either while attending emergency rooms (ERs) with an asthma attack or through daily registries and contact telephone numbers of patients attending the ERs with an asthma attack. From May 2019, eligible patients (Table 2) were provided with a study information sheet on discharge and their parents were contacted to arrange an interview at a hospital consulting room 10-14 days later when they were recruited into the study after providing informed written parental consent and minor assent (8 years and older).

Recruitment – Phase II (peri-COVID-19; March 2021 to March 2022)

The COVID-19 pandemic had a dramatic impact on our ability to recruit children with asthma attacks in the three study hospitals that resulted in the suspension of recruitment in March 2020. This was owing to the reassignment of all 3 hospitals to COVID-19-only activities for prolonged periods and the imposition of a legally enforced lockdown nationally. Figure 2 shows the number of potentially eligible children identified in the 3 study hospitals by month in the period prior to COVID-19 and during the pandemic. There was as a marked reduction in eligible children attending the ERs immediately following the imposition of lockdown on 16th March 2020 and in the following 8 months. In the period following the initial lockdown and other mitigation strategies to reduce transmission of SARS-CoV-2, within the sample of asthmatic children recruited into Phase I of the study, there was evidence of dramatic reduction in health service attendance rather than changes in the incidence of attacks [19]. To allow continued recruitment, a second phase (Phase II) of the study was started with eligibility criteria modified to include children and adolescents aged 5-17 years registered as having asthma in patient registries at the study hospitals in Cuenca and Portoviejo (Quito was not included in Phase II because of logistic issues in that specific setting). The child's parent was contacted by telephone and asked about the child's current asthma symptoms. Children with current symptoms were considered eligible. Eligibility criteria for Phase I and II are shown in Table 2.

Table 2 Eligibility criteria for entry into Phase I and Phase II of study

Characteristic	Phase I	Phase II
Setting/study population	Public hospital ERs in Quito, Cuenca, and Portoviejo	Public hospital registry of patients with asthma in Cuenca and Portoviejo
Inclusion criteria	1. Aged 5-17 years<br>**2. Acute asthma attack attending ERs at public hospitals**<br>3. Living within 12 km of public hospital<br>4. Informed written consent from parents<br>5. Minor assent from children >=8 years	1. Aged 5-17 years<br>**2. Wheeze within the last 6 months**<br>3. Living within 12 km of public hospital<br>4. Informed written consent from parents<br>5. Minor assent from children >=8 years
Exclusion criteria	1. Other chronic disease<br>2. Living >12 km from public hospital	1. Other chronic disease<br>2. Living >12 km from public hospital

Data collection

A study manager supervised day-to-day activities of a dedicated teams (physician and nurse) of trained study personnel based in each of the public hospitals. Each participant was followed up for 12 months in Phase I and at least 6 months in Phase II. Standardized procedures were used throughout (Figure 2). Subjects were evaluated at monthly intervals using face-to-face and telemonitoring for follow-up. Both phases include face-to-face evaluations (evaluations at baseline, 2, 4, 6, 9, and 12 months in Phase I; baseline, 6, and 12 months in Phase II). Between March 2020 and March 2021, all face-to-face evaluations in Phase I were changed to telemonitoring apart from the final evaluation at 12 months which remained face-to-face, where possible. Final face-to-face evaluations were done in March-April 2021 on participants who had completed 12 months of follow-up between June 2019 and February 2020. The site of face-to-face evaluations was changed to either a clinic or home visit depending on the preference of the child's parents. Study procedures were the same in both phases of the study (Figure 2) with the following exceptions: 1) procedures requiring face-to-face evaluations at 6 and 12 months in Phase I (C-ACT, and PAQLQ) were dropped in Phase

For peer review only - http://bmjopen.bmj.com/site/about/guidelines.xhtml

II and the remaining instruments were administered by telemonitoring; 2) blood samples (baseline, 6, and 12 months) are being collected from those recruited into Phase II of the study for measurement of anti-SARS-CoV-2 IgG antibodies; and 3) measurement of lung function and fractional exhaled nitric oxide was suspended from March 2020 because of risks from potentially hazardous infectious aerosols until a time when this procedure can be done safely. Medications (inhaled salbutamol and fluticasone) were provided free of charge to participants when prescribed by a hospital physician and there was no medication in the hospital pharmacy. The study team ensured adequate inhaler technique during face-to-face clinic or home visits. Study personnel did not interfere with treatment indications of hospital staff.

Figure 2. Study procedures during baseline evaluation and follow-up in Phases I (A) and II (B) of study.

BaselineQ – General questionnaire based on Phase II of the International Study of Asthma and Allergies in Childhood; AFCQ- Asthma Family Costs Questionnaire; NAKQ – Newcastle Asthma Knowledge Questionnaire; ACT – Asthma Control Questionnaire (>11 years); C-ACT – Child Asthma Control Questionnaire (>=12 years); ARQ – Asthma Recurrence Questionnaire; FeNO – fractional exhaled nitric oxide. Follow-up in Phase I was for 12 months and in Phase II for a minimum of 6 months.

Questionnaires at baseline and follow-up

The following questionnaires were used as indicated in Figure 2; i) asthma questionnaire to collect information on history of asthma symptoms, treatment and management, and potential risk factors for asthma attacks. This questionnaire has been previously adapted from ISAAC Phase II and extensively field-tested for children and adolescents with asthma [13,26]; ii) Asthma Recurrence Questionnaire (ARQ) - questionnaire to monitor recurrence of asthma symptoms and monitor treatment during follow-up [13], and including symptoms and diagnosis of COVID-19 in Phase II; iii) Childhood Asthma Control Test (C-ACT) [45], completed by the child and parent for children aged <12 years or the ACT [46] for those aged ⩾12 years; iv) Pediatric Asthma

Quality of Life Questionnaire (PAQLQ) [47]; v) Newcastle Asthma Knowledge Questionnaire (NAKQ) [48,49] validated in a Spanish-speaking populations; and vi) Asthma Family Cost Questionnaire (AFCQ) [50]. Data collection is summarized in Table 3.

Table 3 Data collection at baseline and during follow-up in Phases I and II of study

Data collected	Baseline	Month 6	Month 12	Monthly
Asthma diagnosis	I/II			
History of asthma symptoms	I/II			
Current asthma symptoms	I/II	I/II	I/II	I/II
ER visits/ hospitalizations	I/II	I/II	I/II	I/II
Asthma medications	I/II	I/II	I/II	I/II
Asthma control (C-ACT/ACT)	I/II	(I)/II	(I)/II	
Asthma quality of life (PAQLQ)	I/II	(I)/II	(I)/II	
Asthma knowledge (NAKQ)	I			
Lung function<br>Reversibility<br>FeNO<br>Anti-SARS-CoV-2 IgG antibodies	I/(II)<br>I/(II)<br>I/II<br>II	(I/II)<br>(I/II)<br>(I)/II<br>II	(I)<br>(I)<br>(I)/II<br>II	
COVID-19 symptoms or diagnosis	II	II	II	II

FeNO – fractional exhaled nitric oxide. () – data collection where possible

Lung function test and Fractional Exhaled Nitric Oxide (FeNO)

For peer review only - http://bmjopen.bmj.com/site/about/guidelines.xhtml

Lung function testing is being done according to American Thoracic Society (ATS) criteria using a portable ultrasonic spirometer (EasyOne® device, ndd Medical Technologies). Global Lung Function Initiative (GLI-2012) reference values will be used to calculate z-scores depending on age, sex, height, and ethnicity [51]. Lung function is being measured before and before and after 200 ug salbutamol administered through a spacer with a positive test for airways reactivity defined as an increase in FEV_1 of ≥12%. Fractional exhaled nitric oxide (FeNO) is being measured in parts per billion using NObreath (Bedfont Scientific, UK). As a consequence of lockdown and mitigation strategies to prevent transmission of SARS-CoV-2, measurement of lung function and FeNO was suspended in March 2020. FeNO measurement, a low effort procedure that carries a minimal risk of generating aerosols [52,53], was resumed in March 2021 for 12-month evaluations in Phase I and baseline evaluations in Phase II. FeNO is being done following the recommendations of the manufacturer to minimize potential risks [54]. Spirometry, which often induces cough, will be resumed, only if the procedure can be done without risk to staff, patients, and their families.

Blood samples

Blood sample are being taken at baseline, 6 and 12 months in Phase II. Blood is drawn from an antecubital vein into a 4 mL plastic tube containing EDTA as anticoagulant (Vacutainer, BD). After centrifugation, plasma is stored at -20C until analysis. Levels of IgG specific for SARS-CoV-2 are being measured using a validated enzyme linked immunosorbent assay [55].

Biosafety precautions relating to the COVID-19 pandemic

Personnel gathering data during face-to-face visits have been trained to use appropriate personal protective equipment according to international guidelines [56–58]. Immediately prior to a home or clinic visit, the child's carer is telephoned about potential symptoms or a COVID-19 diagnosis among family members or close contacts – if any symptoms are reported the visit is rescheduled after 2 weeks.

Sample size considerations

For peer review only - http://bmjopen.bmj.com/site/about/guidelines.xhtml

A sample size of 450 patients was proposed for Phase I, based on a binary outcome variable of any recurrence of an asthma attack within 12 months or so. We used an estimated recurrence risk of about 50% during 6 months follow-up from a similar population in Ecuador [13]. The study should collect between 10 and 16 outcome events per each potential explanatory variable for robust results (i.e. for at least 80% power, equivalent to a type 2 error of less than 20% as proven by simulation studies). Assuming 10 explanatory variables are considered, in the worst-case scenario, 10×16=160 such events would be required for the upper limit of 16 events for each explanatory variable. Given a 50% risk of recurrence, a minimum of 160×2=320 subjects will be needed. Allowing for 20% attrition/loss during follow up given the longitudinal nature of the study, we needed approximately 400 patients which we set more conservatively at 450 [59–62]. However, we do not expect a multivariable model to exceed 10 predictors. There is the possibility of differences between populations recruited during the 2 phases of the study. If this is not the case, an indicator variable which annotates the two phases will be added to the set of predictors and 450 patients would still allow at least 80% power under the conditions described. For example, if 11 predictors are considered, 352 patients would be required or a total of 440 allowing for 20% attrition. If there are substantial differences between subjects recruited in the two phases, analyses will consider up to 10 events per explanatory variable requiring 100 events per phase among 200 participants followed-up or 250 recruited (allowing for 20% attrition). The latter scenario will require recruitment of a total of 500 during the two phases, a target that we are confident we can achieve.

The definition of an asthma attack in both phases of the study is an episode of acute wheezing requiring an unscheduled visit to a health professional (including visits to ERs) and which is controlled by β_2-agonist bronchodilator treatment. The original definition for an asthma attack in Phase I that included ER attendance had to be modified in the light of changes in health seeking behaviour caused by the COVID-19 pandemic and fear of contagion in hospitals and other acute health facilities [19]. Potential explanatory variables include age, gender, ethnicity, socioeconomic status, medication use, asthma attacks and use of systemic corticosteroids in year prior to

For peer review only - http://bmjopen.bmj.com/site/about/guidelines.xhtml

recruitment, asthma control test score, and study centre. Given changes to the Phase I protocol, an additional variable (for recruitment from ERs vs. hospital lists) will be considered as an effect modifier. Sub-group analyses of Phase II will include SARS-CoV-2 seropositivity as an explanatory variable. Secondary outcomes will asthma control score and economic costs to the patient's family.

Statistical analyses

The statistical models to be used by study objective are summarized in Table 1. The primary outcome will be any asthma attack recurrence during 12 months of follow-up in Phase I and at least 6 months in Phase II. Logistic and linear regression, and survival analyses will be used. For the latter, if no re-attendance related to an asthma attack occurred during the follow-up period, the patient is considered (right) censored. The general assumption for time-to-event (survival analysis) is that the censoring is non-informative, i.e. it is independent of the events (asthma attacks). Under this assumption, the results should not be qualitatively different from those inferred from a logistic regression. Odds ratios and/or hazard ratios and their corresponding p-values and 95% CIs will measure the associations between asthma attack recurrence and explanatory variables, and their statistical significance and uncertainty. The Cox proportional hazard (PH) semi-parametric technique allows no assumption to be made on the baseline hazard; nevertheless, proportionality of the hazards should hold. The latter assumption can be tested on the basis of Shoenfeld residuals after fitting models to the data. If unsatisfactory, alternative parametric models will be considered after understanding the distributional assumptions on the data such that the baseline hazard function is modelled appropriately. After exploring the data as described above, a forward-backward elimination survival analysis would be applied to determine the most parsimonious model for an adjusted multivariable model; that is the model with the least number of predictors yet explaining most of the variability in the data. Penalized-likelihood criteria such as AIC or BIC will be used to choose between non-nested models – nevertheless based on complete observations only.

For peer review only - http://bmjopen.bmj.com/site/about/guidelines.xhtml

Hosmer-Lemeshow measure of goodness of fit would be employed for logistic regression models with a p-value of less than 0.05 indicating a poor fit to the data. Model validation will be done by splitting the data set into training-testing subsets (logistic regression setting). Cross validation techniques will be used to understand the prediction power of the corresponding logistic regression model using area under the receiver operator curve, prior to propose the model entering the clinical practice with the view of elaborating a prognostic model. In survival setting it is also possible that the precise time of the attacks may be missed but recorded as between two time points. This would fall into interval censored data as an alternative to survival setting in which the precise time of the event is required.

Multiple recurrences of asthma attacks within the follow-up period will generate a longitudinal binary outcome, with multiple records for each participant over follow-up. The hierarchical structure of the data requires special inferential techniques which account for the two sources of variability: that of between participants and within participants. Three different modelling approaches will be considered: generalized estimation equation, mixed models, and random effects survival models. The first approach provides population-average estimates, the second provides subject-specific estimates while the third is also known as shared frailty survival model. A frailty is a latent random effect which enters multiplicatively on the hazard function and accounts for the multiple failures setting and/or for the clustered aspect of the data.

Complete data analyses will be done under missing completely at random assumptions. However, patterns in missing data will be explored and methods to evaluate potential impact of missing data on estimates will be used as appropriate such as multiple imputations using chained equations (MICE) accompanied by a sensitivity analysis to the missing data assumptions [63–65]. Analyses will use STATA (StataCorp. 2017. Stata Statistical Software: Release 15. College Station, TX: StataCorp LLC) or R (R Core Team (2013).

Discussion

For peer review only - http://bmjopen.bmj.com/site/about/guidelines.xhtml

The prevalence of childhood asthma has increased in many LMICs over the past 50 or so years where asthma attacks are a growing burden to health systems [2]. There are limited data on determinants of asthma attacks in children and adolescents from LMIC settings [18], and a need for prospective studies to identify risk factors for asthma attacks so that the limited resources available in low-resource settings can be focused on those children most likely to suffer recurrent attacks to reduce morbidity and economic costs to health systems and patients' families. The Asthma ATTACK study aims to identify risk factors associated with asthma attacks among children and adolescents attending emergency rooms in public hospitals in three Ecuadorian cities.

This study provides a range of climatic settings in Ecuador from high altitude (Cuenca and Quito) to sea level (Portoviejo). Study activities were interrupted in March 2020 by the COVID-19 pandemic resulting in a suspension of recruitment in hospital ERs owing to dramatic changes in access to and use of health facilities. As a consequence, the study protocol was modified to allow re-initiation of recruitment and also the evaluation of the effect of exposures to SARS-CoV-2 on risk of asthma attacks and symptoms in children. The study will help fill an unmet knowledge gap on the effects of SARS-CoV-2 on pediatric asthma, particularly from low-resource LMIC settings [19].

Study limitations

A potential limitation is recruitment of sufficient subjects with an acute asthma attack attending emergency rooms at the three public hospitals and ensuring monthly follow-up in Phase I of the study. The COVID-19 pandemic had a major impact on health seeking behaviours while mitigation strategies to control transmission of the novel coronavirus, SARS-CoV-2, resulted in marked declines in the circulation of respiratory viruses that are considered to cause a high proportion of asthma attacks [66]. A shift to telemonitoring during the COVID-19 pandemic helped ensure almost complete follow-up of those recruited prior to March 2020, although procedures requiring the presence of the patient or that generated potentially hazardous aerosols (e.g. spirometry) had to be suspended. Declines in circulating respiratory viruses [67] because of mitigation strategies against COVID-19 could reduce the number of

For peer review only - http://bmjopen.bmj.com/site/about/guidelines.xhtml

recurrence events and hence the power of the study. Phase II is recruiting known asthmatic children and adolescents with recent asthma symptoms and following them-up for asthma attacks for at least 6 months. The change in criteria should allow sufficient subjects to be recruited given the impact of COVID-19 pandemic on health attendance behaviours, although continuing mitigation measures may reduce recurrence. Follow-up in Phase II is being done through telemonitoring and a few scheduled face-to-face visits either to the study clinic or the participant's home depending on individual preferences. Other potential limitations include missing of events although monthly contacts should minimize these. Significant losses to follow-up that could lead to selection bias are being minimized by regular contacts with caregivers including home visits, and confounding should be reduced by the collection of data on a wide variety of known potential risk factors and confounders including treatments received and adherence to those treatments.

Ethics and dissemination

Ethical approval for Phase I was obtained from the Ethics Committee of the Hospital General Docente de Calderon (CEISH-HGDC 2019-001) and for Phase II from the Ecuadorian Ministry of Public Health COVID-19 Ethics Committee (MSP-CGDES-2021-0041-O N° 096-2021). The study results will be disseminated through presentations at conferences and to key stakeholder groups including policy makers, postgraduate student theses, peer-review publications, and on a study website.

References

1. Reddel HK, Busse WW, Pedersen S, Tan WC, Chen Y-Z, Jorup C, et al. Should recommendations about starting inhaled corticosteroid treatment for mild asthma be based on symptom frequency: a post-hoc efficacy analysis of the START study. Lancet. 2017;389: 157–166.

2. Pearce N, Aït-Khaled N, Beasley R, Mallol J, Keil U, Mitchell E, et al. Worldwide trends in the prevalence of asthma symptoms: phase III of the International Study of Asthma and Allergies in Childhood (ISAAC). Thorax. 2007;62: 758–766.

3. Hasegawa K, Bittner JC, Nonas SA, Stoll SJ, Watase T, Gabriel S, et al. Children and Adults With Frequent Hospitalizations for Asthma Exacerbation, 2012-2013:

A Multicenter Observational Study. J Allergy Clin Immunol Pract. 2015;3: 751–8.e1.

4. Deschildre A, Pichavant M, Engelmann I, Langlois C, Drumez E, Pouessel G, et al. Virus-triggered exacerbation in allergic asthmatic children: neutrophilic airway inflammation and alteration of virus sensors characterize a subgroup of patients. Respir Res. 2017;18: 191.

5. Murray CS, Foden P, Sumner H, Shepley E, Custovic A, Simpson A. Preventing Severe Asthma Exacerbations in Children. A Randomized Trial of Mite-Impermeable Bedcovers. Am J Respir Crit Care Med. 2017;196: 150–158.

6. Taquechel K, Diwadkar AR, Sayed S, Dudley JW, Grundmeier RW, Kenyon CC, et al. Pediatric Asthma Health Care Utilization, Viral Testing, and Air Pollution Changes During the COVID-19 Pandemic. J Allergy Clin Immunol Pract. 2020;8: 3378–3387.e11.

7. Bønnelykke K, Sleiman P, Nielsen K, Kreiner-Møller E, Mercader JM, Belgrave D, et al. A genome-wide association study identifies CDHR3 as a susceptibility locus for early childhood asthma with severe exacerbations. Nat Genet. 2014;46: 51–55.

8. Fischer GB, Sarria EE, Camargos P, Mocelin HT, Soto-Quiroz M, Cruz AA, et al. Childhood asthma in low and middle-income countries: Where are we now? Paediatr Respir Rev. 2019;31: 52–57.

9. Carroll WD, Wildhaber J, Brand PLP. Parent misperception of control in childhood/adolescent asthma: the Room to Breathe survey. Eur Respir J. 2012;39: 90–96.

10. Vervloet M, van Dijk L, Spreeuwenberg P, Price D, Chisholm A, Van Ganse E, et al. The Relationship Between Real-World Inhaled Corticosteroid Adherence and Asthma Outcomes: A Multilevel Approach. J Allergy Clin Immunol Pract. 2020;8: 626–634.

11. Fleming L. Asthma exacerbation prediction: recent insights. Curr Opin Allergy Clin Immunol. 2018;18: 117–123.

12. Nunes C, Pereira AM, Morais-Almeida M. Asthma costs and social impact. Asthma Res Pract. 2017;3: 1.

13. Ardura-Garcia C, Arias E, Hurtado P, Bonnett LJ, Sandoval C, Maldonado A, et al. Predictors of severe asthma attack re-attendance in Ecuadorian children: a cohort study. Eur Respir J. 2019;54. doi:10.1183/13993003.02419-2018

14. Smiley M, Sicignano N, Rush T, Lee R, Allen E. Outcomes of follow-up care after an emergency department visit among pediatric asthmatics in the military health system. J Asthma. 2016;53: 816–824.

For peer review only - http://bmjopen.bmj.com/site/about/guidelines.xhtml

15. Beck AF, Simmons JM, Huang B, Kahn RS. Geomedicine: area-based socioeconomic measures for assessing risk of hospital reutilization among children admitted for asthma. Am J Public Health. 2012;102: 2308–2314.

16. Zipkin R, Schrager SM, Nguyen E, Mamey MR, Banuelos I, Wu S. Association between pediatric home management plan of care compliance and asthma readmission. J Asthma. 2017;54: 761–767.

17. Moncrief T, Beck AF, Simmons JM, Huang B, Kahn RS. Single parent households and increased child asthma morbidity. J Asthma. 2014;51: 260–266.

18. Ardura-Garcia C, Stolbrink M, Zaidi S, Cooper PJ, Blakey JD. Predictors of repeated acute hospital attendance for asthma in children: A systematic review and meta-analysis. Pediatr Pulmonol. 2018;53: 1179–1192.

19. Ochoa-Avilés AM, Ochoa-Avilés C, Morillo-Argudo DA, Molina-Cando MJ, Rodas-Espinoza CR, Chis Ster I, et al. Impact of COVID-19 pandemic on asthma symptoms and management: A prospective analysis of asthmatic children in Ecuador. World Allergy Organ J. 2021;14: 100551.

20. McGeachie MJ, Yates KP, Zhou X, Guo F, Sternberg AL, Van Natta ML, et al. Patterns of Growth and Decline in Lung Function in Persistent Childhood Asthma. N Engl J Med. 2016;374: 1842–1852.

21. Forno E, Gogna M, Cepeda A, Yañez A, Solé D, Cooper P, et al. Asthma in Latin America. Thorax. 2015;70: 898–905.

22. Alith MB, Gazzotti MR, Nascimento OA, Jardim JR. Impact of asthma control on different age groups in five Latin American countries. World Allergy Organ J. 2020;13: 100113.

23. Gold LS, Thompson P, Salvi S, Faruqi RA, Sullivan SD. Level of asthma control and health care utilization in Asia-Pacific countries. Respir Med. 2014;108: 271–277.

24. Sastre J, Fabbri LM, Price D, Wahn HU, Bousquet J, Fish JE, et al. Insights, attitudes, and perceptions about asthma and its treatment: a multinational survey of patients from Europe and Canada. World Allergy Organ J. 2016;9: 13.

25. Reddel HK, Sawyer SM, Everett PW, Flood PV, Peters MJ. Asthma control in Australia: a cross-sectional web-based survey in a nationally representative population. Medical Journal of Australia. 2015. pp. 492–496. doi:10.5694/mja14.01564

26. Ardura-Garcia C, Vaca M, Oviedo G, Sandoval C, Workman L, Schuyler AJ, et al. Risk factors for acute asthma in tropical America: a case-control study in the City of Esmeraldas, Ecuador. Pediatric Allergy and Immunology. 2015. pp. 423–430. doi:10.1111/pai.12401

For peer review only - http://bmjopen.bmj.com/site/about/guidelines.xhtml

27. Cruz AA, Souza-Machado A, Franco R, Souza-Machado C, Ponte EV, Santos PM, et al. The Impact of a Program for Control of Asthma in a Low-Income Setting. World Allergy Organization Journal. 2010. pp. 167–174. doi:10.1097/wox.0b013e3181dc3383

28. Cusack RP, Satia I, O'Byrne PM. Asthma maintenance and reliever therapy: Should this be the standard of care? Ann Allergy Asthma Immunol. 2020;125: 150–155.

29. Abrams EM, Becker AB, Szefler SJ. Paradigm Shift in Asthma Therapy for Adolescents: Should It Apply to Younger Children as Well? JAMA pediatrics. 2020. pp. 227–228.

30. Abrams EM, Shaker M, Greenhawt M, Fernandes RM, Sinha I. Treatment of mild-to-moderate asthma in childhood and adolescence in 2021. Lancet Respir Med. 2021;9: 443–445.

31. Reddel HK, FitzGerald JM, Bateman ED, Bacharier LB, Becker A, Brusselle G, et al. GINA 2019: a fundamental change in asthma management: Treatment of asthma with short-acting bronchodilators alone is no longer recommended for adults and adolescents. Eur Respir J. 2019;53. doi:10.1183/13993003.01046-2019

32. Neffen H, Chahuàn M, Hernández DD, Vallejo-Perez E, Bolivar F, Sánchez MH, et al. Key factors associated with uncontrolled asthma – the Asthma Control in Latin America Study. Journal of Asthma. 2020. pp. 113–122. doi:10.1080/02770903.2018.1553050

33. Liu S, Zhi Y, Ying S. COVID-19 and Asthma: Reflection During the Pandemic. Clin Rev Allergy Immunol. 2020;59: 78–88.

34. Wang Y, Ao G, Qi X, Xie B. The association between COVID-19 and asthma: A systematic review and meta-analysis. Clinical & Experimental Allergy. 2020. pp. 1274–1277. doi:10.1111/cea.13733

35. Hartmann-Boyce J, Gunnell J, Drake J, Otunla A, Suklan J, Schofield E, et al. Asthma and COVID-19: review of evidence on risks and management considerations. BMJ Evid Based Med. 2020. doi:10.1136/bmjebm-2020-111506

36. Peters MC, Sajuthi S, Deford P, Christenson S, Rios CL, Montgomery MT, et al. COVID-19-related Genes in Sputum Cells in Asthma. Relationship to Demographic Features and Corticosteroids. Am J Respir Crit Care Med. 2020;202: 83–90.

37. Yamaya M, Nishimura H, Deng X, Sugawara M, Watanabe O, Nomura K, et al. Inhibitory effects of glycopyrronium, formoterol, and budesonide on coronavirus HCoV-229E replication and cytokine production by primary cultures of human nasal and tracheal epithelial cells. Respir Investig. 2020;58: 155–168.

For peer review only - http://bmjopen.bmj.com/site/about/guidelines.xhtml

38. Schultze A, Douglas I. COVID-19 and inhaled corticosteroids—another piece in an expanding puzzle. The Lancet Respiratory Medicine. 2021. pp. 674–675. doi:10.1016/s2213-2600(21)00076-x

39. Papadopoulos NG, Mathioudakis AG, Custovic A, Deschildre A, Phipatanakul W, Wong G, et al. Childhood asthma outcomes during the COVID-19 pandemic: Findings from the PeARL multi-national cohort. Allergy. 2021;76: 1765–1775.

40. Kaye L, Theye B, Smeenk I, Gondalia R, Barrett MA, Stempel DA. Changes in medication adherence among patients with asthma and COPD during the COVID-19 pandemic. J Allergy Clin Immunol Pract. 2020;8: 2384–2385.

41. Romero NC, Cisneros-Caceres MJ, Granadillo E, Aragao E, Romero-Sandoval A, Barbosa C, Barreto de Oliveira AL, et al. Health workers' perspectives on asthma care coordination between primary and specialised healthcare in the COVID-19 pandemic: a protocol for a qualitative study in Ecuador and Brazil. BMJ Open 2021;11:e052971.

42. Instituto Nacional de Meteorología e Hidrología. Anuario Meteorológico Nro. 53-2013. Quito, Ecuador: INAMHI; 2015.

43. Ministerio de Salud Pública. Manual del modelo de atención integral del sistema nacional de salud familiar comunitario e intercultural (MAIS-FCI). Ecuador: MAIS-FCI; 2012 [cited 22 Apr 2021]. Available: https://es.scribd.com/document/338645477/Manual-MAIS-MSP12-12-12-pdf

44. Lucio R, Villacrés N, Henríquez R. Sistema de salud de Ecuador. Salud pública de México. 2011;53: s177–s187.

45. Liu AH, Zeiger R, Sorkness C, Mahr T, Ostrom N, Burgess S, et al. Development and cross-sectional validation of the Childhood Asthma Control Test. J Allergy Clin Immunol. 2007;119: 817–825.

46. Nathan RA, Sorkness CA, Kosinski M, Schatz M, Li JT, Marcus P, et al. Development of the asthma control test☆A survey for assessing asthma control. Journal of Allergy and Clinical Immunology. 2004. pp. 59–65. doi:10.1016/j.jaci.2003.09.008

47. Juniper EF, Guyatt GH, Feeny DH, Ferrie PJ, Griffith LE, Townsend M. Measuring quality of life in children with asthma. Qual Life Res. 1996;5: 35–46.

48. Fitzclarence CA, Henry RL. Validation of an asthma knowledge questionnaire. J Paediatr Child Health. 1990;26: 200–204.

49. Praena Crespo M, Lora Espinosa A, Aquino Llinares N, Sánchez Sánchez AM, Jiménez Cortés A. [The Spanish version of the Newcastle Asthma Knowledge Questionnaire for parents of children with asthma (NAKQ). Transcultural adaptation and reliability analysis]. An Pediatr . 2009;70: 209–217.

For peer review only - http://bmjopen.bmj.com/site/about/guidelines.xhtml

50. Franco R, Nascimento HF, Cruz AA, Santos AC, Souza-Machado C, Ponte EV, et al. The economic impact of severe asthma to low-income families. Allergy. 2009;64: 478–483.

51. Quanjer PH, Stanojevic S, Cole TJ, Baur X, Hall GL, Culver BH, et al. Multi-ethnic reference values for spirometry for the 3-95-yr age range: the global lung function 2012 equations. Eur Respir J. 2012;40: 1324–1343.

52. Association for Respiratory Technology & Physiology. ARTP COVID-19 GROUP: Practical guidance for increasing Lung Function and Sleep activity during COVID-19 Endemic Phase. Aug 2020 [cited 25 Jan 2021]. Available: https://www.artp.org.uk/write/MediaUploads/Standards/COVID19/ARTP_COVID_Practical_Innovations_Vers1.2_final.pdf

53. PCRS. Position Statement Diagnostic work up of the patient presenting with respiratory symptoms during the COVID-19 pandemic. In: The Primary Care Respiratory Society [Internet]. 17 Sep 2020 [cited 25 Jan 2021]. Available: https://www.pcrs-uk.org/sites/pcrs-uk.org/files/resources/COVID19/Diagnosis-asthma-COPD-during-COVID-19-v2-17-September-2020.pdf

54. Coronavirus & the 2nd Generation NObreath - Update. In: NObreath [Internet]. [cited 25 Jan 2021]. Available: https://www.nobreathfeno.com/coronavirus-and-the-2nd-generation-nobreath-update/

55. Guevara Á, Vivero S, Nipaz V, Guaraca V, Coloma J. A Low-Cost SARS-CoV-2 rRBD ELISA to Detect Serostatus in Ecuadorian Population with COVID-19. Am J Trop Med Hyg. 2021. doi:10.4269/ajtmh.20-1420

56. AAAAI. Spirometry during COVID-19. In: American Academy of Allergy, Asthma & Immunology [Internet]. 23 Apr 2020 [cited 25 Jan 2021]. Available: https://www.aaaai.org/ask-the-expert/spirometry

57. Stanojevic S, Beaucage F, Comondore V, Faughnan M, Kovesi T, McCoy C, et al. Resumption of pulmonary function testing during the post-peak phase of the COVID-19 pandemic. Canadian Journal of Respiratory, Critical Care, and Sleep Medicine. 2020. pp. 156–159. doi:10.1080/24745332.2020.1796211

58. McGowan A, Sylvester K, Burgos F, Boros P, de Jongh F, Kendrick A, et al. Recommendation from ERS Group 9.1 (Respiratory function technologists/Scientists) Lung function testing during COVID-19 pandemic and beyond. ; 2020 [accessed 2020 Aug 31]. In: European Respiratory Society [Internet]. [cited 18 Jul 2020]. Available: https://ers.app.box.com/s/zs1uu88wy51monr0ewd990itoz4tsn2h

59. Vittinghoff E, McCulloch CE. Relaxing the rule of ten events per variable in logistic and Cox regression. Am J Epidemiol. 2007;165: 710–718.

60. Peduzzi P, Concato J, Kemper E, Holford TR, Feinstein AR. A simulation study of

the number of events per variable in logistic regression analysis. J Clin Epidemiol. 1996;49: 1373–1379.

61. Concato J, Peduzzi P, Holford TR, Feinstein AR. Importance of events per independent variable in proportional hazards analysis. I. Background, goals, and general strategy. J Clin Epidemiol. 1995;48: 1495–1501.

62. Peduzzi P, Concato J, Feinstein AR, Holford TR. Importance of events per independent variable in proportional hazards regression analysis. II. Accuracy and precision of regression estimates. J Clin Epidemiol. 1995;48: 1503–1510.

63. White IR, Royston P, Wood AM. Multiple imputation using chained equations: Issues and guidance for practice. Stat Med. 2011;30: 377–399.

64. White IR, Royston P. Imputing missing covariate values for the Cox model. Stat Med. 2009;28: 1982–1998.

65. Royston P, Altman DG. External validation of a Cox prognostic model: principles and methods. BMC Med Res Methodol. 2013;13: 33.

66. Ahanchian H, Jones CM, Chen YS, Sly PD. Respiratory viral infections in children with asthma: do they matter and can we prevent them? BMC Pediatr 2012;12:147.

67. Influenza and other respiratory viruses. https://www.paho.org/en/topics/influenza-and-other-respiratory-viruses

Author statement: Study design – PJC, AAC, NR, MB, MP, CA-G; study conduct – DM, SM-B, AO, MEC, CR, AM, KA, AR, CF, JA, KS; manuscript drafting – PJC, DM, SM-B; statistical analysis plan – ICS; manuscript editing and review – all authors.

Funding: This work is based on the Asthma Attacks Causes and Prevention Study in Urban Latin America which is funded by the National Institute of Health Research [NIHR] under the Global Health Research Programme [grant 17/63/62]. Additional financial support was provided by the Corporación Ecuatoriana para el Desarrollo de la Investigación y la Academia CEDIA [N° CEPRA-XV-2021-03].

Competing Interests statement: The authors declare no competing interests.

For peer review only - http://bmjopen.bmj.com/site/about/guidelines.xhtml

Patient and public involvement statement: There was no patient or public involvement in the design or conduct of this study. Results of tests are being provided at the time of testing and the study population is being informed of study results through periodic leaflets, a website, social media texts and videos, and study group online meetings.

For peer review only - http://bmjopen.bmj.com/site/about/guidelines.xhtml

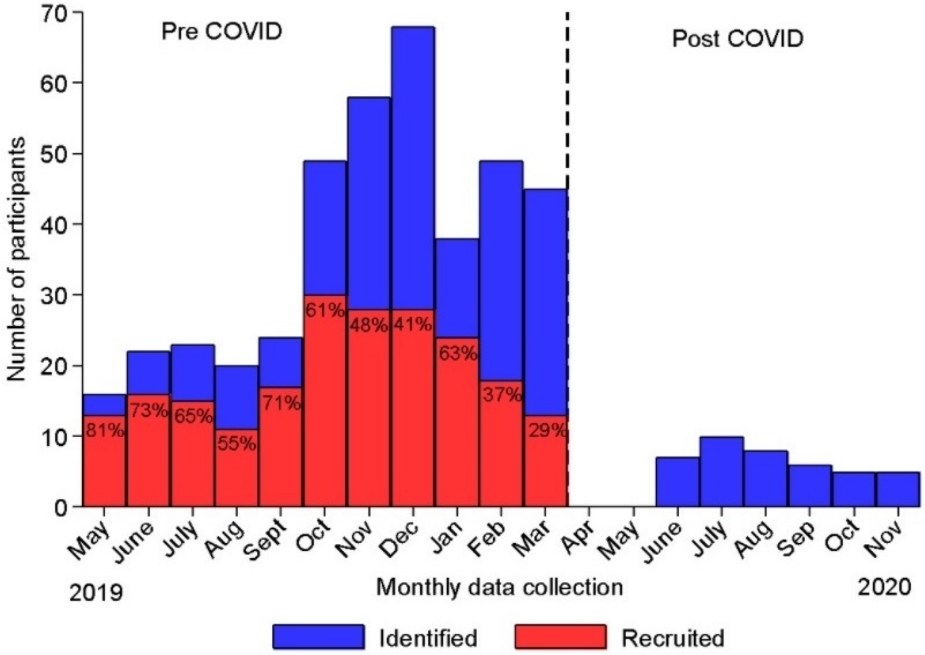

Figure 1

152x107mm (144 x 144 DPI)

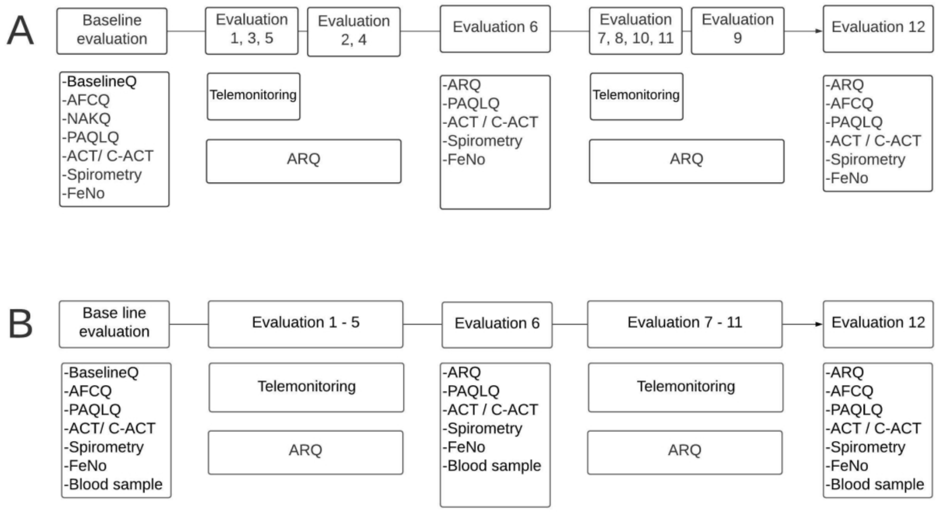

Figure 2

408x217mm (160 x 160 DPI)
